# 5′ Untranslated Region Elements Show High Abundance and Great Variability in Homologous ABCA Subfamily Genes

**DOI:** 10.3390/ijms21228878

**Published:** 2020-11-23

**Authors:** Pavel Dvorak, Viktor Hlavac, Pavel Soucek

**Affiliations:** 1 Department of Biology, Faculty of Medicine in Pilsen, Charles University, 32300 Pilsen, Czech Republic; 2 Biomedical Center, Faculty of Medicine in Pilsen, Charles University, 32300 Pilsen, Czech Republic; viktor.hlavac@szu.cz (V.H.); pavel.soucek@szu.cz (P.S.); 3 Toxicogenomics Unit, National Institute of Public Health, 100 42 Prague, Czech Republic

**Keywords:** 5′ untranslated region, cis-acting elements, ABC transporters, ABCA subfamily, bioinformatics

## Abstract

The 12 members of the ABCA subfamily in humans are known for their ability to transport cholesterol and its derivatives, vitamins, and xenobiotics across biomembranes. Several ABCA genes are causatively linked to inborn diseases, and the role in cancer progression and metastasis is studied intensively. The regulation of translation initiation is implicated as the major mechanism in the processes of post-transcriptional modifications determining final protein levels. In the current bioinformatics study, we mapped the features of the 5′ untranslated regions (5′UTR) known to have the potential to regulate translation, such as the length of 5′UTRs, upstream ATG codons, upstream open-reading frames, introns, RNA G-quadruplex-forming sequences, stem loops, and Kozak consensus motifs, in the DNA sequences of all members of the subfamily. Subsequently, the conservation of the features, correlations among them, ribosome profiling data as well as protein levels in normal human tissues were examined. The 5′UTRs of ABCA genes contain above-average numbers of upstream ATGs, open-reading frames and introns, as well as conserved ones, and these elements probably play important biological roles in this subfamily, unlike RG4s. Although we found significant correlations among the features, we did not find any correlation between the numbers of 5′UTR features and protein tissue distribution and expression scores. We showed the existence of single nucleotide variants in relation to the 5′UTR features experimentally in a cohort of 105 breast cancer patients. 5′UTR features presumably prepare a complex playground, in which the other elements such as RNA binding proteins and non-coding RNAs play the major role in the fine-tuning of protein expression.

## 1. Introduction

The proteins in the ABC (ATP-binding cassette) family can be found in every group of living organisms, from bacteria to primates, and are generally known for their ability to translocate a wide range of substrates across extracellular as well as intracellular biomembranes [1,2]. Typically, ABC transport proteins contain two nucleotide-binding domains and two transmembrane domains. ABC proteins are organized as full- or half-transporters in eukaryotes. The products of half-transporters have to homodimerize or heterodimerize to create a functional transporter. Forty-eight ABC protein-coding genes, which have been described in the human genome, are divided into seven subfamilies according to the similarity in their amino acid (aa) sequences and organization of protein domains [3]. The ABCA subfamily is represented by 12 full transporters, which belong to the largest molecules among ABC proteins, with a median of 1925 aa. They have been reported to play important roles in the transport of cholesterol and its derivatives, as well as some vitamins and xenobiotics [4,5]. Several members of the ABCA subfamily have been causatively linked to a diverse set of human inborn diseases such as familial high-density lipoprotein (HDL) deficiency (*ABCA1*), neonatal surfactant deficiency (*ABCA3*), degenerative retinopathies (*ABCA4*), and congenital keratinization disorders (*ABCA12*) [6]. Gene expression studies conducted in our laboratories have demonstrated associations of intra-tumoral transcript levels of several ABCA genes with the response of patients to oncological therapy or disease-free survival [7,8,9,10,11]. Their roles in cancer progression and metastasis attributed mainly to lipid trafficking are a matter of intensive research [4,12]. Phylogenetic analyses suggest that current ABCA genes evolved by many duplication and loss events from a common ancestor gene [6,13,14]. *ABCA5*-related genes (*ABCA5/6/8/9/10*), which evolved from the *ABCA5* gene by duplications, form a cluster on the q-arm of the human chromosome 17 (17q24). The remaining ABCA genes are dispersed on six other human chromosomes [5,15].

**Figure 1:**
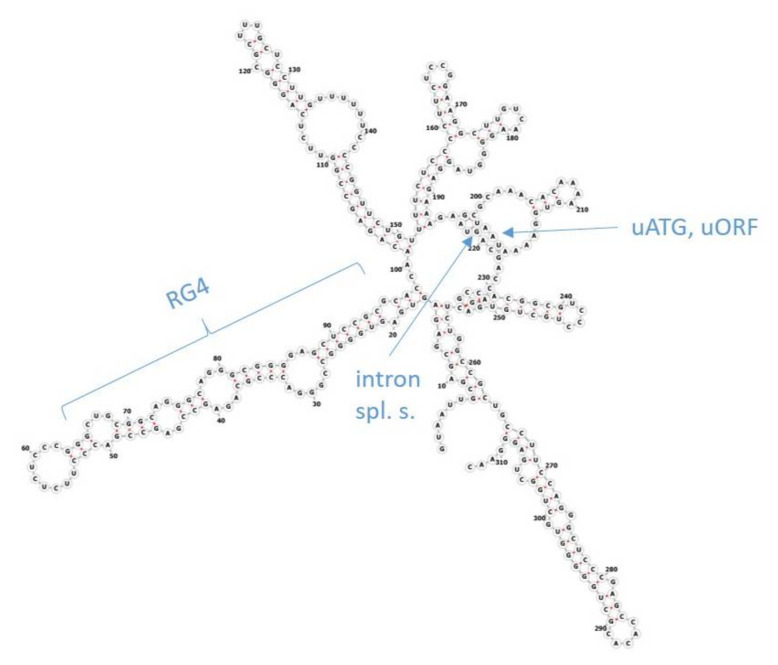
Secondary structure of the 5′UTR of the human *ABCA1* gene. The positions of the features influencing the initiation of translation are depicted; intron spl. s., intron splice-sites; RG4, RNA G-quadruplex-forming sequence; uATG, upstream ATG codon; uORF, upstream open-reading frame.

Gene expression at the protein level does not reflect the mRNA level in normal human tissues perfectly, not only in the case of ABC genes [4,16]. The reason for this difference is believed to lie in post-transcriptional regulation. Regulation of the initiation of translation has been implicated as the major mechanism in this complex process. Several features in the 5′ untranslated regions (5′UTR, also leader sequence) of genes, such as the length of 5′UTRs, upstream ATG start codons (uATG), upstream open-reading frames (uORF), introns, RNA G-quadruplex-forming sequences (RG4), diverse secondary structures like stem loops as well as the Kozak consensus motif in the vicinity of start codons, act as *cis*-acting regulatory factors (Figure 1) [17,18]. RNA structures such as stem loops and RG4s, as well as uORFs and uATGs, mainly inhibit translation. RNA modifications, or RNA-binding proteins (RBP) and long non-coding RNAs (lncRNA) that interact with RNA binding sites, as well as the Kozak motif, can additionally stimulate translation initiation. It is still not clear how the actions of these elements interact, when multiple factors are present together, or if some of them have a superior role. Several highly conserved elements have been revealed in our recent bioinformatics study focusing on the 5′UTRs of the human *ABCA1* gene and its vertebrate orthologs [19]. The 5′UTRs of the other ABCA subfamily genes have not yet been studied in detail. Mapping of the 5′UTR features that are known to have the potential to regulate translation, among the whole subfamily, is addressed in the current work. Those interpreting the significance of new mutations and polymorphisms can take our findings into consideration.

## 2. Results

### 2.1. Alignment and Phylogenetic Tree of 5′UTRs of Human ABCA Genes

**Figure 2:**
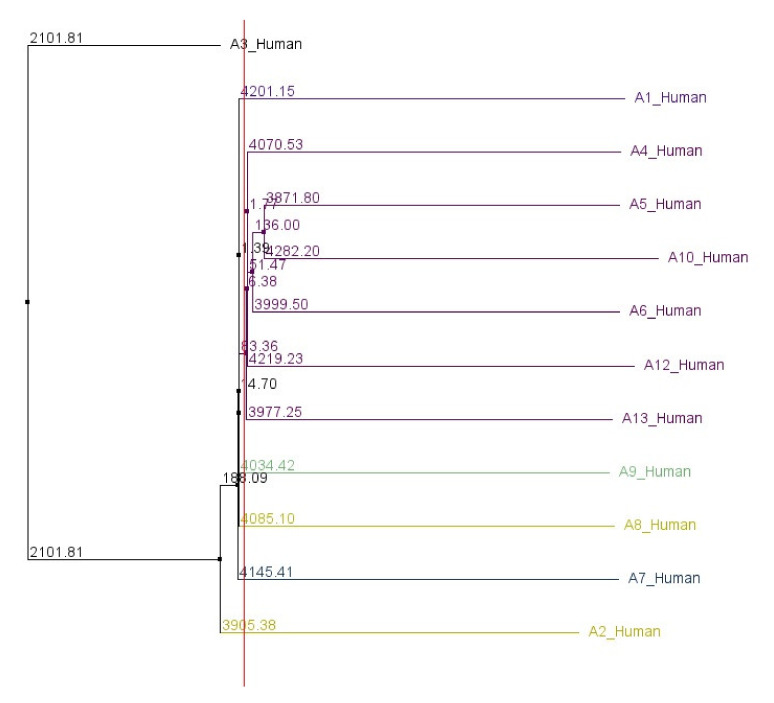
Phylogenetic analysis of 5′UTR sequences of 12 human ABCA genes. The neighbor-joining tree was constructed using DNA model distance measure of Clustal Omega multiple sequence alignment.

Based on the alignment analyses of the individual human ABCA genes with their vertebrate orthologs, we were able to define the sub-regions of the 5′UTRs showing a very high level of conservation. The names, IDs and basic characteristics of the transcripts analyzed are disclosed in Table S1 and positions of these sub-regions are recorded in Table S2. Notably, we did not find any comparable highly conserved sub-regions in the alignment of all human ABCA genes together. Figures S1 and S2 document the alignment of human *ABCA1* and its orthologs for ClustalO and Mafft algorithms, respectively. Figures S3 and S4 show the alignment of all human ABCA genes in a similar manner. A phylogenetic tree, based on the 5′UTR sequences of all human ABCA genes, was constructed and is shown in Figure 2.

The order in which 5′UTRs of ABCA genes are described in the following sections reflects their relationships calculated in the phylogenetic analysis.

### 2.2. ABCA3

The ABCA3-201 transcript, coding for the main functional isoform, with 33 exons is 6602 bp long. It has the second longest 5′UTR among ABCA genes spanning 694 bps. The 5′UTR is divided into four parts by the three introns—Intron 1–2 (10718 bp), Intron 2–3 (890) and Intron 3–4 (1960). The most conserved region was localized to −519 to −503 from the start ATG codon of the main ORF (sATG). Four uATGs were described at the following positions: 1) −525, 2) −498, 3) −329, and 4) −262. The third and fourth uATGs are conserved in primates (cat. 1), the first uATG in placental mammals (cat. 3) and the second uATG in placental mammals as well as reptiles and birds (cat. 4). The second and fourth showed weak contexts, the first and third adequate contexts. High TIS scores were calculated for the first and second, and middle scores for the third and fourth. Two probable uORFs were predicted: one is 150 nt long (starting at −525) and the second overlapping with the main ORF (starting at −262); and one RG4-forming sequence at −608 to −576. Five stem loops were predicted in this region. The ABCA3 protein was found to be expressed in many human tissues (42 out of 45 tissues tested). The Mode expression score was Medium, enhanced in e. g. brain, glands, lung, testis and spleen. Table 1 discloses an overview of the 5′UTR features for the 12 human ABCA genes. A more detailed overview, including the positions of all features studied, can be viewed in Table S2.

### 2.3. ABCA1

The human ABCA1-202 transcript is 10,408 bp long and has 50 exons. The 5′UTR region covers 313 bps and is divided into two parts by one intron (Intron 1–2), which is the longest among ABCA genes with 24,163 bp. The most conserved sub-region of the 5′UTR was localized to −119 to −59. One uATG was found at −89. This uATG is highly conserved among vertebrates (category 5 = placental mammals + Reptiles and birds + coelacanth/ray-finned fishes); however, it shows a weak flanking sequence context and middle TIS score. One uORF starting at this uATG and overlapping with the main ORF was predicted. The most probable RG4-forming sequence was placed to −251 to −218. Six stem loops were predicted in this region. The ABCA1 protein was found to be expressed in all human tissues (45 tissues). The mode expression score was medium, and the expression was high in lung, stomach, and placenta.

### 2.4. ABCA4

The transcript ABCA4-201 is 7328 bp long and has 50 exons. The 5′UTR is 103 bp long. The sub-region −65 to −50 was determined to be the most conserved. One uATG (−62) is conserved in placental mammals (cat. 3); however, it has a weak context and low TIS score. No probable uORF or RG4-forming sequence was detected. Two stem loops were predicted to this region. The ABCA4 protein was found to be expressed only in the retina with a high score.

### 2.5. ABCA5

The transcript ABCA5-201 is 8252 bp long and contains 39 exons. The 5′UTR covers 97 bps and is interrupted by one intron-Intron 1–2 (12,621 bp). Only a very short sequence −7 to −2 was identified as the most conserved. No uATG, uORF, or RG4-forming sequences were detected and four stem loops were predicted in this region. The ABCA5 protein was found to be expressed in all human tissues (45 tissues). The mode expression score was high.

### 2.6. ABCA10

The human *ABCA10* gene has no known orthologs in ray-finned fishes. The ABCA10-201 transcript is 6362 bp long and has 40 exons. It has the longest 5′UTR among ABCA genes encompassing 910 nts. Three introns (Intron 1–2, 15,668 bp; Intron 2–3, 1295 bp; Intron 3–4, 1615 bp) are annotated within the 5′UTR. The most conserved sub-region was determined to −459 to −368. Fourteen uATGs (1. −755, 2. −722, 3. −709, 4. −645, 5. −625, 6. −574, 7. −571, 8. −482, 9. −464, 10. −279, 11. −265, 12. −197, 13. −169, 14. −56) were discovered. The seventh uATG is present only in humans (cat. 0), the first, second, third, fifth, sixth and eighth are conserved within primates (cat. 1), the ninth to fourteenth (last five) are conserved within placental mammals (cat. 3), and the fourth within placental mammals as well as reptiles and birds (cat. 4). The third, fifth, sixth, tenth, and fourteenth uATGs show weak contexts; the first, second, fourth, seventh, and eighth adequate contexts; the ninth, eleventh, twelfth, and thirteenth strong contexts. The fourteenth uATG shows a low TIS score, the fourth and eleventh high TIS scores, and the others middle TIS scores. Six uORFs (start positions-1. −722, 2. −625, 3. −482, 4. −279, 5. −65, 6. −197) were predicted; 33, 117, 42, 33, 171, and 39 bp long, respectively. Interestingly, the sATG of this transcript shows only an adequate flanking sequence context. No probable RG4-forming sequence and five stem loops were predicted in this region. The ABCA10 protein was found to be expressed in many human tissues (40/45). The mode expression score was medium.

### 2.7. ABCA6

The human *ABCA6* gene has no known orthologs in Sauropsida (reptiles and birds) and ray-finned fishes. The transcript ABCA6-201 is 5321 bp long and has 39 exons. The 5′UTR is 196 nt long and divided into two parts by Intron 1–2 (996 bp). The most conserved sub-region is located to −156 to −63. Two uATGs were found within the region (−144 and −32). The first uATG is conserved within placental mammals (cat. 3) and shows an adequate context and middle TIS score; the second is conserved only in primates (cat. 1) and has also an adequate context, but low TIS score. Notably, the sATG of this transcript shows only a weak flanking sequence context. No probable uORF or RG4-forming sequence was detected. Three stem loops were predicted in this region. The ABCA6 protein was found to be expressed in all human tissues (31 tissues tested). The Mode expression score was Low, enhanced in e. g. adipose tissue, bone marrow, brain, esophagus, gallbladder, liver, ovary, testis, and urinary bladder.

### 2.8. ABCA12

The ABCA12-201 transcript is 9298 bp long and has 53 exons. The 5′UTR spans the first 418 nts. Three separate sub-regions showed the highest level of conservation. Four uATGs (1. −398, 2. −333, 3. −330, 4. −115) were described. The first and third uATGs are conserved within primates (cat. 1), the second and fourth within placental mammals as well as reptiles and birds (cat. 4). The second uATG shows a weak context and the others adequate contexts; all show middle TIS scores. One 69 nt long uORF was predicted starting from −398. No probable RG4-forming sequence was found and six stem loops were predicted in this region. The ABCA12 protein was found to be expressed in many human tissues (17/31). The mode expression score was medium, enhanced in duodenum, kidney, ovary, and small intestine.

### 2.9. ABCA13

The ABCA13-204 transcript is 17,188 bp long and has 62 exons. The 5′UTR is the smallest among ABCA genes and has only 26 nts. No uATG, uORF or probable RG4-forming sequences were located within the 5′UTR. RNA expression was found in many human tissues, enhanced in bone marrow; however, protein expression has not been annotated yet.

### 2.10. ABCA9

The human *ABCA9* gene has no known orthologs in Sauropsida (reptiles and birds) and ray-finned fishes. The ABCA9-201 transcript is 6377 bp long and has 39 exons. One 9726 bp long intron (Intron 1-2) is annotated within the 75 nt long 5′UTR. Two sub-regions fulfilled the criteria for the most conserved ones. Three uATGs (1. −70, 2. −58, 3. −54) were found; the first and third are conserved within placental mammals (cat. 3) and second only within primates (cat. 1). The first and second show adequate context and third weak context; however, all have low TIS scores. Two uORFs (1. −70, 2. −54) were described, the first 33 and second 39 nt long. No probable RG4-forming sequence was detected and two stem loops were predicted in this region. The ABCA9 protein was found to be expressed in all human tissues (45). The mode expression score was high.

### 2.11. ABCA8

The human *ABCA8* gene has no known orthologs in Sauropsida (reptiles and birds) and ray-finned fishes. The ABCA8-208 transcript is 6002 bp long and has 40 exons. Intron 1-2 (5746 bp) and intron 2-3 (7272) divide the 340 nt long 5′UTR into three parts. One sub-region (−234 to −180) displayed the highest level of conservation. Six uATGs (1. −265, 2. −243, 3. −152, 4. −149, 5. −135, 6. −79) were recognized; all of them are present only in humans (cat. 0) with the exception of the second one, which can also be found in other primates (cat. 1). All uATGs show adequate contexts and middle TIS scores with the exceptions of the second one, which shows a high TIS score, and the sixth one, which shows a low TIS score. Two uORFs were predicted; the first is 39 nt long and starts from the second uATG and the second 75 nt long from the sixth uATG. No probable RG4-forming sequence was detected and three stem loops were predicted in this region. The ABCA8 protein was found to be expressed in some human tissues (14/45). The mode expression score was low, enhanced in adrenal gland, testis, ovary, liver, and adipose tissue.

### 2.12. ABCA7

The length of the ABCA7-201 transcript is 6815 bp and it has 47 exons. The 5′UTR spans 227 nt with one annotated intron-intron 1-2 (1028 bp). One sub-region (−137 to −73) shows the highest level of conservation. One uATG (−103), which is conserved in placental mammals (cat. 3) and has an adequate context and middle TIS score, was found. One uORF overlapping with the main ORF was predicted to start from this uATG. One probable RG4-forming sequence was placed to −217 to −158. Three stem loops were predicted in this region. The ABCA7 protein was found to be expressed in many human tissues (29/45). The mode expression score was low, enhanced only in bone marrow and spleen.

### 2.13. ABCA2

The ABCA2-202 transcript is 8103 bp long and contains 49 exons. The 5′UTR is 97 bp long. The most conserved sub-region was localized to −65 to −12. One uATG (−88) is conserved within primates and rodents (cat. 2); it has an adequate context, however, a low TIS score. Prediction programs found one probable 66 nt long uORF starting at the uATG and one RG4-forming sequence (−81 to −34). Two stem loops were described within this region. The ABCA2 protein was found to be expressed in almost all human tissues lacking only in adipose tissue (44 out of 45). The mode expression score was medium, enhanced only in brain.

### 2.14. Descriptive Statistics Shows High Abundance and Great Variability of 5′UTR Features

Among the 12 ABCA genes, the minimum 5′UTR length is 26 nts (*ABCA13*), maximum length 910 (*ABCA10*) and mean/average (M) 291 (median (Mdn) 212 and mode (Mo) 97). The minimum number of uATGs is 0 (*ABCA5* and *ABCA13*), maximum 14 (*ABCA10*) and M = 3 (Mdn = 2 and Mo = 1). The minimum number of uORFs is 0 (*ABCA4*, *ABCA5*, *ABCA6*, and *ABCA13*), maximum 6 (*ABCA10*) and M = 1 (Mdn = 1 and Mo = 1). The minimum number of 5′UTR introns is 0 (*ABCA2, ABCA4*, *ABCA12*, and *ABCA13*), maximum 3 (*ABCA3* and *ABCA10*) and M = 1 (Mdn = 1 and Mo = 1). The minimum 5′UTR intron length is 890 nts, maximum 24,163 and M = 7208 (Mdn = 5746 and Mo not applicable). Only four genes have one RG4 each (*ABCA1*, *ABCA2*, *ABCA3*, and *ABCA7*). The minimum number of 5′UTR stem loops is 0 (*ABCA13*), maximum 6 (*ABCA1* and *ABCA12*) and M = 3 (Mdn = 3 and Mo = 2). The minimum number of all 5′UTR elements is 0 (*ABCA13*), maximum 22 (*ABCA10*) and M = 8 (Mdn = 6 and Mo = 6). Table 2 presents the summary of the 5′UTRs’ descriptive statistics.

### 2.15. Positive Correlations among 5′UTR Features

**Figure 3:**
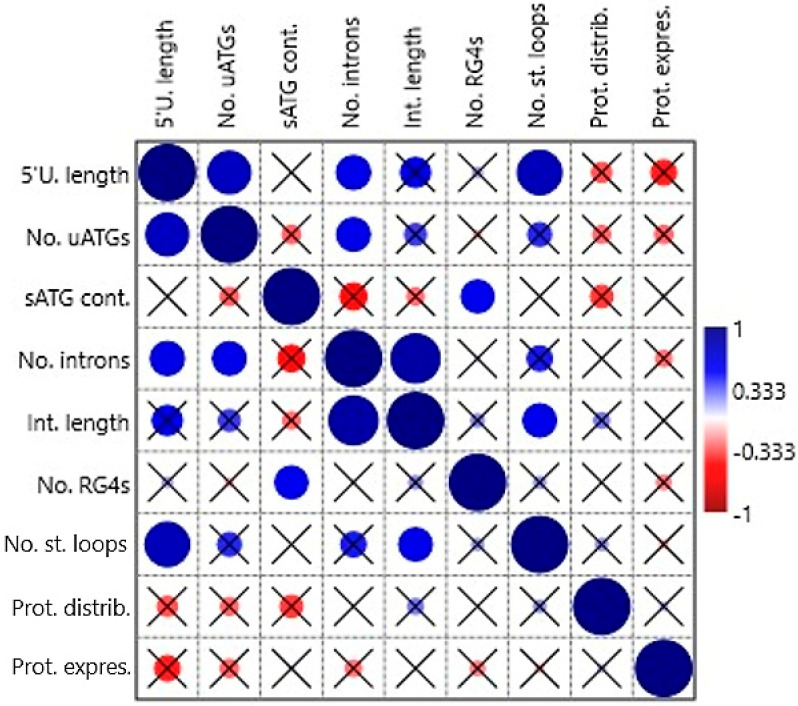
Correlation table plot. The circles represent the Spearman’s *rs* correlation coefficients on a given scale; if the relevant *p* value is >0.05 the circle is crossed. 5′U., 5′UTR; Cont., context; Distrib., distribution; Expres., expression; Int., intron; No., number; Prot., protein; RG4, RNA G-quadruplex; sATG, start ATG of the main ORF; St., stem; uATG, upstream ATG.

Correlations among the following independent variables: 5′UTR length, no. of uATGs, sATG flanking sequence context, no. of 5′UTR introns, length of all 5′UTR introns in a gene, presence of RG4-forming sequence, no. of stem loops, protein tissue distribution, and protein mode expression score were tested by non-parametric tests (Spearman’s rs and Kendall’s tau) in the first correlation analysis. Some positive correlations were found to be statistically significant (Table S3 and Figure 3). The variable 5′UTR length correlated with No. of uATGs (*p* = 0.004 and 0.006, respectively), no. of 5′UTR introns (0.037/0.016), and no. of stem loops (0.002/0.004). Furthermore, no. of uATGs correlated with no. of 5′UTR introns (0.041/0.022), sATG flanking sequence context with the presence of RG4-forming sequence (0.046/0.010), no. of 5′UTR introns with Length of all 5′UTR introns in a gene (<0.001/<0.001), and no. of stem loops with length of all 5′UTR introns in a gene (0.042/0.011). The variables protein tissue distribution and protein mode expression score did not correlate significantly with any other variable.

**Figure 4:**
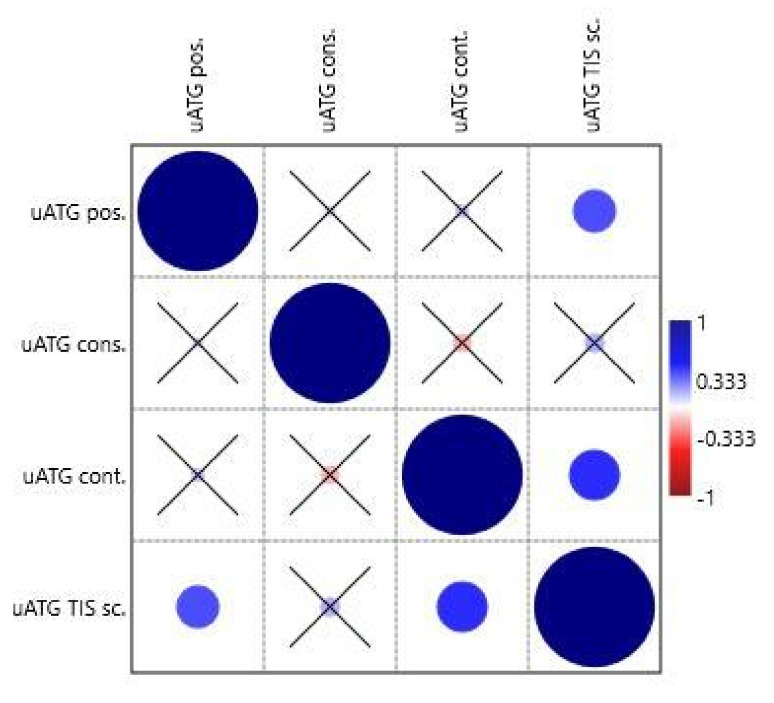
Correlation table plot. The circles represent the Spearman’s *rs* correlation coefficients on a given scale; if the relevant *p* value is > 0.05 the circle is crossed. uATG cons., uATG conservation; uATG cont., uATG flanking sequence context; uATG pos., uATG position (from transcription start site); uATG TIS sc., uATG TIS score (from NetStart).

The special features of uATGs-uATG position (from transcription start site, TSS), uATG conservation, uATG flanking sequence context, and uATG TIS score were tested similarly in the second correlation analysis. The variable uATG TIS score correlated positively with uATG position (0.034/0.031) and uATG flanking sequence context (0.011/0.003) (Table S4 and Figure 4).

### 2.16. Ribo-Seq Data Confirmed Translation at Some of the Predicted uORFs

**Figure 5:**
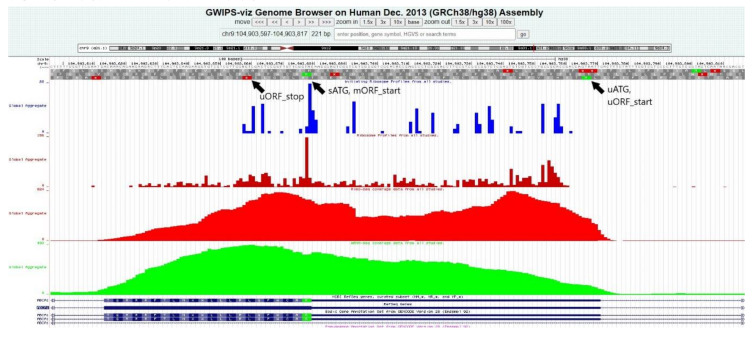
Ribosome profiling analysis in the GWIPS-viz browser, an example of the *ABCA1* gene. High concentrations of ribosomes can be seen at the sites of the predicted upstream ORF and beginning of the main ORF. Blue histograms represent Initiating Ribosome (P-site) Profiles from all studies, dark red—Elongating Ribosome (A-site) Profiles from all studies, red—Ribo-seq coverage data from all studies (Elongating Ribosomes—Footprints), and green—mRNA-seq coverage data from all studies (mRNA-seq Reads). mORF, main open reading frame; sATG, start ATG of the main ORF; uATG, upstream ATG; uORF, upstream open reading frame.

The analysis of the Ribo-seq coverage data confirmed a high concentration of ribosomes within some of the regions where probable uORFs were predicted by the bioinformatics algorithms. The uORFs in the genes *ABCA1*, *ABCA3*, *ABCA7*, and *ABCA8* displayed a high level of ribosome coverage. Notably, a significant ribosome coverage peak was also detected at the site of the first uATG of *ABCA6* (−144), which was not connected to any uORF. An example of the Ribo-seq data analysis in the GWIPS-viz browser is disclosed in Figure 5 (on a case of the *ABCA1* gene); all results regarding uORFs are summarized in Table 3.

### 2.17. Thirteen Single Nucleotide Variants in 5′UTRs of ABCA Genes Were Found in Breast Cancer Patients

Thirteen single nucleotide variants (SNV) in 5′UTRs of main functional isoforms of ABCA genes were found within our cohort of 105 breast cancer patients by targeted sequencing. These SNVs are located within 5′UTRs of *ABCA1*, *ABCA3*, *ABCA7*, and *ABCA10*. An overview of these SNVs is presented in Table 4. One variant in *ABCA10* (rs1238052530) is changing the eighth uATG to GTG and therefore disrupting the start of the third uORF. Eight other variants are located within uORFs (five synonymous changes, two nonsynonymous, and one frameshift) and one within the most conserved region. The location of all 13 SNVs in relation to the 5′UTR features of ABCA genes is visualized in Figure S5.

## 3. Discussion

Our bioinformatics study focused on the 12 members of the human ABCA gene subfamily. These homologous genes are known for their principal role in lipid trafficking and homeostasis; however, other biological functions such as the involvement in signaling pathways activated by lipids and support of tumor progression are discussed in the recent literature. A comprehensive analysis of their 5′UTR sequences, in view of the features known to be involved in translation regulation, was addressed in the current study. We aimed to answer the question if the incidence of these 5′UTR features correlates clearly with protein expression in this group. Since ABCA genes lie behind several human diseases, both inborn and acquired, this information is important for the interpretation of the newly found mutations and polymorphisms in the clinical as well as research settings. Moreover, because similar studies on the gene family level are still missing, we also aimed at a comparison of our results to current knowledge based on the whole-genome level studies, which is addressed in this section.

### 3.1. Phylogenetic Tree

Phylogenetic analyses of human ABCA genes were performed by several previous studies. Some of them are based on the alignment of the nucleotide sequences or amino acid sequences of nucleotide-binding domains [3,20], the others on the alignment of the full length amino acid sequences [6,13]. Pairwise comparison of the amino acid sequences of all human ABCA members revealed homologies ranging from 28% (ABCA8/ABCA12) to 72% (ABCA8/ABCA9) [20]. The previous studies suggested that all ABCA genes have evolved from a primordial ancestor gene. Furthermore, they demonstrated that the human ABCA1, 2, 3, 4, 7, and 12 transporters cluster in a subgroup, distinct from ABCA5, 6, 8, 9, and 10. The ABCA5-related transporters share strikingly high overall amino acid sequence homologies but differ significantly from other members [3,6,13,20]. In contrast, our phylogenetic analysis, based on the comparison of nucleotide sequences of ABCA 5′UTRs, shows that the 5′UTRs of ABCA2 and ABCA3 cluster distinctly from the rest of the 5′UTRs. These discrepancies suggest that the evolution of 5′UTRs is shaped by different pressures independently from the other gene regions.

### 3.2. 5′UTR Length

The average/median length of 5′UTRs is 291/212 nts in the human ABCA genes; however, there is a great variation among the 12 members. The smallest 5′UTR is annotated to *ABCA13* (26 nts) and largest to *ABCA10* (910). In the literature, we find divergent data about the average/median length of human gene 5′UTRs based on the input data and year of study. Pesole and coworkers [21] constructed the first database focusing on the UTR sequences from different eukaryotic taxa and named it UTRdb. The average length of mRNA 5′UTR in humans was calculated to be 210 nts, maximum length 2803 and minimum 18 [22]. Rogozin et al. [23] calculated the average length of human 5′UTRs to be 160 nts (retrieved from EMBL database). In the work of Chen et al. [24], the average/median length of 5′UTRs in humans was calculated to be 254/169 nts from the Ensembl database and 220/160 from the UTRdb database. Recently, Leppek et al. [17] stated that the longest known median length of mRNA 5ʹ UTRs occurs in humans and is 218 nts (based on RefSeq data). We can conclude that the median length of 5′UTRs in the human ABCA subfamily is close to the median length derived from the whole genome data. Indeed, the genome average of 5′UTR lengths is relatively similar across diverse taxonomic classes of eukaryotes, ranging approximately from 100 to 200 nts, while in sharp contrast, the 5′UTR length varies considerably among the genes in a genome, from a few to several thousands of nts, a fact which has been mentioned in several previous studies [17,22,25,26]. Lynch and colleagues [27] suggested that this discrepancy can be explained by random genetic drift and mutational processes that cause stochastic turnover in transcription-initiation sites and premature start codons. Under the simple null model that they presented, natural selection only indirectly influences the lengths of 5′UTRs through the mutational origin of premature initiation codons within the UTR. We further confirmed in this study that the great length variability of 5′UTRs can be observed even in closely related members of a gene subfamily.

### 3.3. uATGs—Number and Conservation

Among human ABCA genes, there are two (17%; *ABCA5* and *ABCA13*) having no uATG; the others (83%) have at least 1 uATG, ranging from 1 to 14 uATGs (*ABCA10*). The median number of uATGs in the ABCA subfamily was computed to be 2 (average is 3 and mode 1), again showing a great variability among individual members. In total, 37 uATGs were found in the subfamily. The median value of uATG conservation was 1 (on a scale of 0–5), minimum 0 and maximum 5. 49% of the uATGs had a conservation value more than 1, that is, conserved in at least one other vertebrate subgroup except primates. Generally, uATGs and uORFs decrease mRNA translation efficiency and may be considered strong negative translational regulatory signals [28,29,30]. One of the approaches to address the issue of the functional significance of uATGs is to examine the evolutionary conservation of these triplets. Churbanov et al. [31] reported that the ATG triplet is conserved to a significantly greater extent than any of the other 63 nucleotide triplets in 5′UTRs of mammalian cDNAs, but not in 3′UTRs or coding sequences, by comparing sequences of human, mouse, and rat orthologous genes. Moreover, they observed that 5′UTRs are significantly depleted in overall ATG content. Approximately 25% of the 5′UTRs analyzed in their work contained at least one conserved uATG. In a similar study performed by Iacono and colleagues [32], uATGs and uORFs were detected in about 44% of 5′UTRs. They also concluded that both uATGs and uORFs are less frequent than expected by chance in 5′UTRs. 24% and 38% of human uATGs and uORFs were evolutionary conserved in all three taxa considered (human, mouse, and rat), respectively. Of the population of human and mouse mRNAs with long 5′UTRs (>60 bases), approximately 55% had at least one uAUG, with about 25% having one or more uORFs (average about 1.9 uORFs) [33]. We found that 5′UTRs of ABCA genes contain above-average numbers of uATGs as well as conserved ones. Approximately half of all uATGs in ABCA are conserved not only within primates, but also in other vertebrate subgroups. Considering this high level of conservation among uATGs, they probably play important biological roles in this subfamily.

### 3.4. uATGs—Flanking Sequence Context

The median value of uATG flanking sequence context in our study was 2 (on a scale of 1–4), minimum 1 and maximum 3. The median value of sATG flanking sequence context was 3, minimum 1 and maximum 3. Churbanov et al. [31] wrote that there was no significant difference between the nucleotide contexts of the conserved and non-conserved uATGs; in both cases, the information content of the uATG context was lower than that of the sATG context. Similarly, the analysis of the oligonucleotide context of uATGs, uORFs and sATGs has shown that a significant preference bias can be observed only for sATGs which, on average, have a much better context than uATGs and uORFs [32]. Our results are also in agreement with Rogozin et al. [23], who found that the presence of ATG triplets in 5′UTR regions of eukaryotic cDNAs correlates with “weaker” contexts of the sATGs. The median sATG context in our study was “strong” (level 3), not “optimal” (level 4). However, newer results on a larger cohorts of annotated transcripts have indicated some specifications, e.g., the proportion of uATGs in optimal contexts for conserved uORFs was noticeably higher than for non-conserved uORFs, while still half the proportion for main ORFs [33].

### 3.5. uORFs

There were eight genes (67%) among ABCA genes having at least one uORF predicted by the prediction software, 16 uORFs in total. The median for the whole subgroup is 1 uORF, minimum 0 and maximum 6 (*ABCA10*). Recently, Johnstone and co-workers [34] published that the human and mouse transcriptomes had similar uORF content (49.5 and 46.1%, respectively), consistent with previous computational estimates. The above-average incidence of uORFs in the ABCA subfamily is obvious from our results. The biological significance of a majority of uORFs is probably not limited to the regulation of translation, as the uORF products—small polypeptides—can play many different roles within the metabolism of complex organisms [35].

### 3.6. 5′UTR Introns

Four ABCA genes (33%) have no 5′UTR intron; the others (67%) have at least one. The median as well as mode number of 5′UTR introns is 1, maximum is 3 (*ABCA3* and *ABCA10*). The median length of 5′UTR introns in ABCA subfamily is 5746 nts, minimum 890 and maximum 24,163 (*ABCA1*). Approximately 35% of human genes have been indicated to harbor introns within 5′UTRs, with median intron size 2643 pb [22,36,37]. A strong barrier against the presence of more than 1 intron in 5′UTRs has also been suggested. In some cases, introns in 5′UTRs were reported to enhance gene expression [38]. Although Cenik et al. [36] found no correlation in 5′UTR intron presence or length with variance in expression across tissues, they observed an uneven distribution of 5′UTR introns amongst genes in specific functional categories. Contrary to the study of Cenik et al. [36], Lim et al. [39] described a strong negative correlation between the number of 5′UTR exon-exon junctions (the junctions between exons after intron removal) and main ORF translation efficiency in the five multicellular eukaryotic species studied (human, mouse, zebrafish, fruit fly and thale cress). We demonstrated an above-average occurrence and length of 5′UTR introns in ABCA genes.

### 3.7. RG4-Forming Sequences

Probable RG4-forming sequences were only predicted in the 5′UTRs of four ABCA genes (33%; *ABCA1*, *ABCA2*, *ABCA3*, and *ABCA7*); three of the sequences were located within the first third of the region. G-quadruplexes (G4) are secondary structures involving four nucleic acid strands that can be adopted by both DNA (DG4) and RNA (RG4) that contain guanine-rich sequences [40,41]. DG4 sequences have been mainly connected with the regulation of transcription and RG4 sequences regulation of translation; however, many other processes such as telomere maintenance, splicing, or RNA localization have also been discussed. Generally, RG4 sequences are believed to play roles as negative regulators of translation, although a positive effect of these structures has also been reported [17,42]. Thus, the regulatory effect of an RG4 sequence is significantly influenced by its position, the surrounding DNA topology, and other factors [43,44]. Huppert et al. [45] mapped the incidence of G4 in human 5′UTRs and calculated that 6.2% of these regions contained putative G4 sequences, with a density of approximately 0.3 per kb. Their data showed the highest density of putative G4 sequences at the 5′-ends of the 5′UTRs, decreasing approximately linearly along the 5′UTR regions. According to the analyses performed by Maizels and Gray [46], in 10–15% of human genes, a G4 motif occurs in the region specifying the 5′UTR of the encoded mRNA. However, the data analyzed in the latter study showed a higher G4 motif frequency at the 3′-ends of the 5′UTRs than at 5′-ends. With a new algorithm named G4Hunter, Bedrat and coworkers [47] found a significantly higher occurrence of G4 sequences in the human genome, with a density of approximately 2.4 per kb. 53.3% of 5′UTRs contained at least 1 G4-forming sequence in their study. In light of the latest results published in the literature, we can conclude that RG4 sequences are present in below-average numbers within the 5′UTRs of ABCA genes.

### 3.8. Stem Loops

The median number of 5′UTR stem loops in ABCA genes was determined to be 3; the mode was 2. The minimum was 0 (*ABCA13*) and maximum 6 (*ABCA1* and *ABCA12*). Stem loops (hairpins) are the most frequent secondary structures, which naturally fold along single-stranded RNA molecules [48]. The strength of their effect on translation is dependent on the location within 5′UTRs as well as their stability; however, they mainly inhibit this process in eukaryotic cells [17]. The mechanism of stem loop influence on translation has been examined in detail in many studies [49,50] and the prevalence of stem loops in 5′UTRs is generally thought to be high [51,52]. However, the precise stem loop incidence in specific gene groups has not been calculated yet and we were not able to compare our results to the results of others. Our work, therefore, brings largely new information on this topic.

### 3.9. Correlations and Influence of 5′UTR Features on Protein Expression

We have demonstrated a great variability in the numbers of the individual 5′UTR features among the genes of the ABCA subfamily. Considering phylogenetic relationships among ABCA genes, there is no clear pattern in these numbers. Some positive correlations among 5′UTR features were found to be statistically significant. Some of these correlations are expectable, such as the correlations among 5′UTR length and No. of uATGs, No. of 5′UTR introns and the no. of stem loops, some are interesting and need further exploration, such as the correlations between the no. of uATGs and the no. of 5′UTR introns or sATG flanking sequence context and the presence of RG4-forming sequence. Notably, the variable uATG TIS score correlated positively, however quite weakly, with uATG position (from transcription start site) and uATG flanking sequence context (from NetStart software). In relation to the canonical cap-dependent translation initiation, a possible influence of the ATG position on the ATG context has been mentioned in several works. Rogozin et al. [23] reported a significant negative correlation between the sATG information content and 5′UTR length for several species. They also concluded that this correlation could be explained by the strong positive correlation between the number of uATGs and the length of the UTR. Lynch and coworkers [27] described a strong distance-dependent gradient of the deficit of uAUGs. Since uATG TIS score and uATG flanking sequence context should be based on the same criteria, we would expect a stronger correlation. However, this correlation was not studied by any other study for comparison. The length of a 5′UTR, which is determined mainly by stochastic events, seems to be the major factor influencing the numbers of the other regulatory features.

Although there is also great variability in the distribution and expression of the ABCA proteins, we did not find any significant correlation that would support a clear connection between the numbers of 5′UTR features and protein expression characteristics. Among the genes which have the smallest numbers of 5′UTR features are *ABCA13*, *ABCA4*, *ABCA2*, and *ABCA5*. *ABCA13* is the least-explored member of the subfamily and the information about its protein distribution and expression in physiological conditions is missing. ABCA4 protein was reported to be expressed only in the retina and the expression level is high. ABCA2 and ABCA5 were found to be expressed in many and all human tissues with mainly medium and high levels, respectively. On the other side, among the genes which show the highest numbers of 5′UTR features are *ABCA10*, *ABCA3*, *ABCA8*, and *ABCA12*. ABCA10, ABCA3, and ABCA12 proteins are all expressed in many human tissues with mainly medium levels. The ABCA8 protein was detected only in several tissues with low expression. In the set of normal human tissues; however, the protein expression levels of the ABCA proteins ranged from low to high for all members, with the exception of ABCA5 (medium to high) and ABCA10 (low to medium). Our results therefore support the view that the great variability in 5′UTR features prepares a complex playground where the other elements such as RNA binding proteins and non-coding RNAs play the major role in the fine-tuning of final protein expressions. Notably, tissue-specific translation repression by miRNAs through binding to uAUGs was demonstrated in Ajay et al. [53].

### 3.10. Limitations

The current study analyzed data available freely on-line and is based on the in silico approaches and analyses. The main disadvantage is the dependence on the quality of the experimental data from the external sources without own experimental validation. The overwhelming amount of biological data, stored and freely available for the research community worldwide; however, calls for in silico filtering of the content as first step. Experimental verification should be performed for the most relevant findings afterwards. We hope to experimentally verify some of the presented results in the future by ourselves or in cooperation with other research groups. Another limitation of the current work lies in the small number of genes studied. However, we aimed to focus on the clinically important and closely related genes of the ABCA subfamily where the available information is scarce. The other ABC gene subfamilies will be added in the ongoing study.

In the support of our gene family approach, it is important to mention that studies which deal with whole genome data sets require some compromises in the data mining. For example, they considered a uORF (or an uAUG) evolutionarily conserved when occurring in an orthologous transcript independently from its sequence, length, and position in the 5′UTR [32]; or, some analyses were performed only on transcripts containing a single uORF [29]. This, of course, simplifies the view on the genome complexity to some degree.

## 4. Materials and Methods

### 4.1. DNA and Protein Sequences from Databases

DNA sequences of the 5′UTRs of the 12 human ABC protein-coding genes grouped together in the ABCA subfamily *ABCA1-10, ABCA12*, and *ABCA13* and amino acid sequences of the whole proteins were downloaded from the Ensembl database (EMBL-EBI; https://www.ensembl.org/index.html) in the FASTA format. The transcripts of the main principal isoforms were chosen for further analyses based on the APPRIS classification system, UniProt annotation score and MANE Select system (description of transcript flags on the Ensembl web pages - https://www.ensembl.org/info/genome/genebuild/transcript_quality_tags.html). A survey of the number of protein-coding isoforms of ABCA genes and genome positions of their 5′UTRs was performed at the beginning. We found that the number of protein-coding isoforms of individual ABCA genes ranges from three to seven transcripts. There are 48 protein-coding transcripts altogether (36 non-principal isoforms). Fifteen out of the 36 non-principal isoforms have their 5′UTRs located within the same genomic regions as the relevant principal isoforms and smaller or equal to the 5′UTRs of principal isoforms in length. Another eight out of the 36 non-principal isoforms do not have 5′UTR sequences annotated. Because of this heterogeneity and inequality of data, we decided to include just one 5′UTR representative for each of the ABCA genes.

5′UTR sequences of orthologous genes from 10 other vertebrate species were selected and downloaded from the same database in relation to each human ABCA gene according to the same criteria. Based on the availability of species in the Ensembl, members of the five subgroups of vertebrates increasingly phylogenetically distant from humans—primates, rodents, other placental mammals, reptiles and birds, and ray-finned fishes—were covered. Table S1 discloses the names, IDs and basic characteristics of all the transcripts downloaded. The numbers, positions and lengths of the 5′UTR introns were also collected.

### 4.2. Alignment Analyses

Multi-sequence alignment analyses were performed with the help of Jalview software (Consortium Project; www.jalview.org/) [54]. Results of the two alignment algorithms–Clustal Omega (EMBL-EBI; https://www.ebi.ac.uk/Tools/msa/clustalo/) and Mafft (EMBL-EBI; https://www.ebi.ac.uk/Tools/msa/mafft/)—were considered. Alignments of individual ABCA genes and orthologs as well as all ABCA genes together were calculated. Figures S1 and S2 show the results of the alignment analysis for the 5′UTRs of the human ABCA1 gene and its vertebrate orthologs with nucleotide percentage identity colored, consensus logos and occupancy score histograms. Figures S3 and S4 show a similar analysis where the 5′UTRs of all 12 human ABCA genes were aligned together. Based on these analyses and criteria, the most conserved subregions within the 5′ UTRs were described in relation to the sATGs. The cut-off values for the identity and occupancy scores were set to 80%.

### 4.3. Phylogenetic Tree

Phylogenetic analysis of 5′UTR sequences of 12 human ABCA genes was performed and visualized with the help of Jalview software. A neighbor-joining tree using DNA model distance measure of Clustal Omega multiple sequence alignment was constructed. Clustal Omega algorithm was set to the default settings.

### 4.4. ATG Analyses

All ATG triplets within the 5′ UTRs of human ABCA genes were found and highlighted in text editor files (MS Word). The positions (relative to the sATGs as well as TSSs) and flanking sequence contexts were recorded. A scale of four categories was applied for the comparison of flanking sequence contexts: (1) weak (NNN(C/U)NNAUG(A/C/U), any sequence lacking both key nucleotides); (2) adequate (NNN(A/G)NNAUG(A/C/U) or NNN(C/U)NNAUGG, only one of these nucleotides is present); (3) strong (NNN(A/G)NNAUGG, only the two important nucleotides are present); and (4) optimal (GCC(A/G)CCAUGG). The scoring system was adopted from Hernandez et al. [55]. A TIS (translation initiation start) score generated by the NetStart prediction server (DTU Health Tech; https://services.healthtech.dtu.dk/service.php?NetStart-1.0) was also recorded for each ATG analyzed. The scores are in the range [0.0, 1.0]; when greater than 0.5 they represent a probable translation start. Based on this definition we further subdivided the TIS scores into three levels: (1) low (less than 0.1), (2) middle (0.1 to 0.5), and (3) high (greater than 0.5). We proposed a scale of six categories for the evaluation of ATG conservation statuses: 0) found only in humans, (1) conserved in primates, (2) in primates and rodents, (3) in primates, rodents and other placental mammals, (4) in placental mammals and reptiles and birds, (5) in placental mammals, reptiles and birds, and coelacanth or ray-finned fishes.

### 4.5. Upstream ORF Analysis

The ORFfinder (NCBI; https://www.ncbi.nlm.nih.gov/orffinder/) online tool was used to search for possible upstream open reading frames (uORFs) within the human 5′UTRs tested. The positions of all uORFs with the same orientation as the main ORFs, in three possible frames, were collected.

### 4.6. RG4 Analyses

The DNA sequences downloaded were screened for the presence of RNA G-quadruplex-forming sequences by the three on-line prediction servers working with different prediction algorithms-G4CatchAll (Doluca lab; http://homes.ieu.edu.tr/odoluca/G4Catchall/) [56], G4RNA Screener (Scott Group Bioinformatics; http://scottgroup.med.usherbrooke.ca/G4RNA_screener/) and QRGS Mapper (Ramapo College; http://bioinformatics.ramapo.edu/QGRS/index.php). The default settings of the programs were not changed and we followed the recommended cut-off levels. We adhered to the following result interpretation: the intersection of the results calculated by the G4CatchAll and G4RNA Screener were recorded as the most probable RG4-forming sequences. In the cases where no intersection of the two programs existed, the intersections of the G4CatchAll and QRGS Mapper or G4RNA Screener and QRGS Mapper were considered instead, and eventually recorded.

### 4.7. RNA Secondary Structure Prediction

RNAfold WebServer (University of Vienna; http://rna.tbi.univie.ac.at/cgi-bin/RNAWebSuite/RNAfold.cgi) was employed to generate the optimal secondary structures for minimum free energy prediction in the form of dot-bracket notation, graphical visualizations colored by base-pairing probabilities and mountain plot representations of the minimum free energy (MFE) structures, the thermodynamic ensembles of RNA structures, and the centroid structures (Figure S5). Equivalent predictions made by RNAstructure Web Servers (Mathews group; http://rna.urmc.rochester.edu/RNAstructureWeb/) were checked for comparison. The number of predicted stem loops within the whole 5′UTRs of the human ABCA genes was compared, counted and recorded.

### 4.8. Protein Expression Level from Databases

The expression of ABCA genes at the protein level was analyzed in the Human Protein Atlas (Consortium Project; http://www.proteinatlas.org) and Expression Atlas (EMBL-EBI; https://www.ebi.ac.uk/gxa/home). The expression of ABCA genes was tested in 45 and 31 normal human tissues in the Human Protein Atlas and Expression Atlas, respectively. The two variables reflecting the overall distribution and expression in human tissues and their following levels were collected: (A) Protein tissue distribution: 1-One tissue/2-Some tissues (less than a half of the tissues studied in the atlases)/3-Many tissues (equal to or greater than half of the tissues)/4-All tissues, and (B) Mode expression score (the most common level of protein expression based on immunohistochemistry scoring): 1-Not detected/2-Low/3-Medium/4-High. The mode expression score was calculated for the set of tissues where protein expression was detected.

### 4.9. Ribosome Profiling from Databases

Ribosome profiling (Ribo-seq) data were explored in the GWIPS-viz browser (http://gwips.ucc.ie/), an online genome browser for viewing ribosome profiling data. These four tracks were considered: initiating ribosome profiles from all studies, ribosome profiles from all studies, Ribo-seq coverage data from all studies and mRNA-seq coverage data from all studies. At a particular site, Ribo-seq coverage was qualitatively evaluated as 1-Low (less than 25%), 2-Medium (between 25% and 75%), or 3-High (more than 75%) in relation to the relevant sATG and mORF.

### 4.10. Statistics

Basic descriptive statistics as well as correlation analyses were computed with the help of the PAST software (University of Oslo; Paleontological Statistics, version 4.03, https://www.nhm.uio.no/english/research/infrastructure/past/). Non-parametric correlation coefficients (Spearman’s *rs* and Kendall’s tau) were calculated for the correlation analyses. Monte Carlo permutation tests were available for all the correlation coefficients. The significance was computed using a two-tailed *t* test with *n – 2* degrees of freedom. A *p* value less than 0.05 was considered statistically significant.

### 4.11. Patient and Sample Characteristics

Our cohort was composed of 105 breast cancer patients of Caucasian origin diagnosed in the Institute for the Care for Mother and Child and Medicon in Prague and Hospital Atlas in Zlin (all in the Czech Republic) during 2006–2013. Patients underwent neoadjuvant cytotoxic therapy with regimens based on 5-fluorouracil/anthracyclines/cyclophosphamide (FAC or FEC) and/or taxanes (*n* = 68) or postoperative adjuvant therapy using the same cytotoxic drugs (*n* = 37). The distribution of molecular subtypes was as follows: Luminal A 16%, Luminal B 24%, and triple negative 60%. These patients constituted the testing set in our previous study conducted by Hlavac et al. [57] and are characterized in detail in the article. Collection of blood samples and DNA Extraction were carried out according to standard procedures and described previously.

### 4.12. Targeted Sequencing

5′UTR sequences of all human ABCA genes were sequenced within a broader set of all exons of 509 genes representing major drug metabolizing and transporting enzymes, nuclear receptors, cell death, chemotherapy target, and signaling pathway genes. The gene panel selection, libraries preparation, sequencing criteria and data analysis, including selection and annotation of variants, were precisely described in Hlavac et al. [57].

## 5. Conclusions

We showed that the great variability among 5′UTR features seen on a whole genome level can be observed even in the group of homologous ABCA subfamily genes. Our phylogenetic analysis, based on the comparison of nucleotide sequences of ABCA 5′UTRs, shows that the 5′UTRs of *ABCA2* and *ABCA3* cluster distinctly from the rest of the 5′UTRs. The 5′UTRs of ABCA genes contain above-average numbers of uATGs, uORFs and 5′UTR introns as well as conserved ones and these elements probably play important biological roles in this subfamily, unlike RG4s. Our work brings largely new information on the numbers of stem loops in 5′UTRs. Some of the positive correlations among 5′UTR features are likely, however, some are interesting and need further exploration, such as the correlations between number of uATGs and number of 5′UTR introns or sATG flanking sequence context and presence of RG4-forming sequence. The lengths of the ABCA 5′UTRs seem to be the major factor influencing the numbers of the other known 5′UTR regulatory elements. Although there is also great variability in the distribution and expression of the ABCA proteins, we did not find any significant correlation between the numbers of 5′UTR features and protein expression characteristics. However, we confirmed a high concentrations of ribosomes at some of the predicted uORFs in the analysis of Ribo-seq data. We further verified the existence of SNVs in relation to the 5′UTR features, predicted within this study, experimentally in our cohort of 105 breast cancer patients. Our results support the view that the other elements such as RNA binding proteins and non-coding RNAs play the major role in protein expression fine-tuning within the complex background of the highly variable 5′UTRs. These findings extend our view on human genome variability and raise new questions for further investigations.

## Figures and Tables

**Table 1:** Overview of the 5′UTR features in human ABCA genes sorted according to their phylogenetic relationships.

DNA/RNA								PROTEIN		
Gene	5′UTR Length [nt]	No. of uATGs	No. of uORFs	No. of 5′UTR Introns	No. of RG4-Forming Seq.	No. of 5′UTR Stem Loops	No. of All 5′UTR Elements	Protein Tissue Distrib.	Protein Mode Expres. Score	Protein Expres. Score Range
*ABCA3*	694	4	2	3	1	5	13	3	3	2–4
*ABCA1*	313	1	1	1	1	6	9	4	3	2–4
*ABCA4*	103	1	0	0	0	2	3	1	4	n.a.
*ABCA5*	97	0	0	1	0	4	5	4	4	3–4
*ABCA10*	910	14	6	3	0	5	22	3	3	2–3
*ABCA6*	196	2	0	1	0	3	6	4	2	2–4
*ABCA12*	418	4	1	0	0	6	10	3	3	2–4
*ABCA13*	26	0	0	0	0	0	0	n.a.	n.a.	n.a.
*ABCA9*	75	3	2	1	0	2	6	4	4	2–4
*ABCA8*	340	6	2	2	0	3	11	2	2	2–4
*ABCA7*	227	1	1	1	1	3	6	3	2	2–4
*ABCA2*	97	1	1	0	1	2	4	3	3	2–4

Abbreviations: Distrib., distribution; Expres., expression; n.a., not available; No., number; seq., sequence; uATG, upstream ATG; uORF, upstream ORF. Protein tissue distribution (in normal human tissues): 1-One/2-Some (less than a half)/3-Many/4-All. Protein expression score: 1-Not detected/2-Low/3-Medium/4-High.

**Table 2:** Descriptive statistics of the 5′UTR features in human ABCA genes.

	No. uATGs	No. uORFs	No. 5′UTR Introns	No. RG4s	No. Stem Loops	No. All 5′UTR Elements
No. genes	12	12	12	12	12	12
Min	0	0	0	0	0	0
Max	14	6	3	1	6	22
Sum	37	16	13	4	41	95
Mean	3	1	1	0	3	8
Variance	15	3	1	0	3	33
Median	2	1	1	0	3	6
Mode	1	1	1	0	2	6

No., number; RG4, RNA G-quadruplex; uATG, upstream ATG; uORF, upstream ORF.

**Table 3:** Summary of the predicted uORFs in ABCA genes with Ribo-seq coverage data.

Gene	No. of uORFs	uORF Start	uORF End	uORF Length (nt)	Ribo-Seq Cov.
*ABCA3*	2	−525	−376	150	3
		−262	>+1	>261	2
*ABCA1*	1	−89	>+1	>87	3
*ABCA4*	0				
*ABCA5*	0				
*ABCA10*	6	−722	−690	33	1
		−625	−509	117	1
		−482	−441	42	1
		−279	−247	33	1
		−265	−95	171	1
		−197	−159	39	1
*ABCA6*	0				
*ABCA12*	1	−398	−330	69	1
*ABCA13*	0				
*ABCA9*	2	−70	−38	33	1
		−54	−16	39	1
*ABCA8*	2	−243	−205	39	3
		−79	−5	75	1
*ABCA7*	1	−103	>+1	>102	3
*ABCA2*	1	−88	−23	66	1

Abbreviations: Cov., coverage; No., number; uORF, upstream ORF; Ribo-seq coverage: 1 = Low, 2 = Medium, 3 = High; All positions are described in relation to the start ATG of the main ORF.

**Table 4:** Overview of single nucleotide variants in 5′UTRs of ABCA genes in 105 breast cancer patients.

Gene	SNV	Position within 5′UTR	Genotypes ^1^			Variation Type and Localization in Relation to 5′UTR Features
			Common Homo-Zygous	Hetero-Zygous	Rare Homo-Zygous	
*ABCA1*	rs1800978	−18	80	24	1	SNV (C > G) within uORF, synonymous change
*ABCA1*	rs1799777	−77–−76	80	25	0	Indel variant (dupC) within uORF, frameshift change
*ABCA1*	rs111292742	−279	97	8	0	SNV (G > C)
*ABCA3*	rs45487892	−67	103	2	0	SNV (G > A) within uORF2, nonsynonymous change
*ABCA3*	rs45518738	−182	104	1	0	SNV (G > A) within uORF2, synonymous change
*ABCA3*	rs146642275	−397	104	1	0	SNV (G > A) within uORF1, synonymous change
*ABCA3*	rs1029783163	−409	104	1	0	SNV (G > A) within uORF1, synonymous change
*ABCA7*	rs182233998	−14	101	4	0	SNV (T > C) within uORF, synonymous change
*ABCA7*	rs3752229	−9	93	12	0	SNV (A > G) within uORF, nonsynonymous change
*ABCA10*	rs1024510317	−89	104	1	0	SNV (G > C)
*ABCA10*	rs9302891	−438	0	14	91	SNV (G > T) within the most conserved region
*ABCA10*	rs1238052530	−482	104	1	0	SNV (T > C) disrupting the start of uORF3 (uATG8 to GTG)
*ABCA10*	rs563620435	−762	104	1	0	SNV (C > A)

Footnote: ^1^ Genotypes do not sum up to 105 due to missing data; SNV, single nucleotide variant.

## Supplementary Materials

Supplementary Materials can be found at https://www.mdpi.com/1422-0067/21/22/8878/s1. **Table S1.** Basic characteristics of the transcripts downloaded and analyzed in the study. **Table S2.** A detailed overview of 5′UTR features in the human ABCA genes. **Table S3.** Correlation tables of the first correlation analysis with Spearman’s rs and Kendall’s tau coefficients and relevant p values. **Table S4.** Correlation tables of the second correlation analysis with Spearman’s rs and Kendall’s tau coefficients and relevant p values. **Figure S1.** Multi-sequence alignment (Clustal Omega algorithm) of the 5′UTRs of the human ABCA1 gene and its vertebrate orthologs with nucleotide percentage identity colored, consensus logos and occupancy score histograms. **Figure S2.** Multi-sequence alignment (Mafft algorithm) of the 5′UTRs of the human ABCA1 gene and its vertebrate orthologs with nucleotide percentage identity colored, consensus logos and occupancy score histograms. **Figure S3.** Multi-sequence alignment (Clustal Omega algorithm) of the 5′UTRs of all 12 human ABCA protein-coding genes with nucleotide percentage identity colored, consensus logos and occupancy score histograms. **Figure S4.** Multi-sequence alignment (Mafft algorithm) of the 5′UTRs of all 12 human ABCA protein-coding genes with nucleotide percentage identity colored, consensus logos and occupancy score histograms. **Figure S5.** The location of 13 SNVs, found in breast cancer patients, in relation to the 5′UTR features of ABCA genes.

## Abbreviations

5′UTR	5′ untranslated region/leader sequence
ABC	ATP-binding cassette
cat.	Category
RG4	RNA G-quadruplex
sATG	Start ATG of the main ORF
TSS	Transcription start site
uATG	Upstream ATG start codon
uORF	Upstream open-reading frames
