# Supplementary material for: 5′ Untranslated Region Elements Show High Abundance and Great Variability in Homologous ABCA Subfamily Genes
Source: Int J Mol Sci. 2020 Nov 23;21(22):8878. doi: 10.3390/ijms21228878 (PMC7700387; doi:10.3390/ijms21228878)
Supplement: Supplementary file 1 [file ijms-21-08878-s001.zip › Figure S5 v1 SNVs versus 5UTR features.pdf]

Figure S5. The location of 13 SNVs, found in breast cancer patients, in relation to the 5’UTR features of ABCA genes.

AAA RNA G quadruplex-forming sequence

TTT Highly conserved sequence

ATG Upstream ATG codon

↓ Intron position

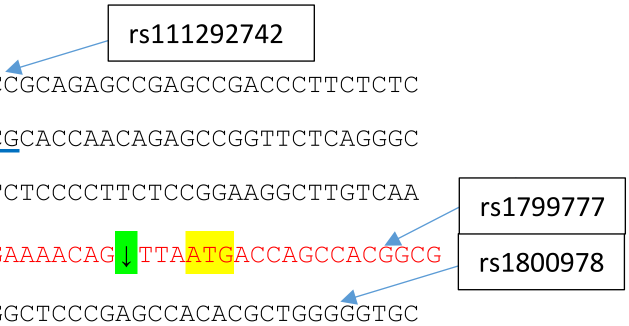

>A1_Human

GTAATTGCGAGCGAGAGTGAGTGGGGCCGGGACCCGCAGAGCCGAGCCGACCCTTCTCTC

CCGGGCTGCGGCAGGGCAGGGCGGGGAGCTCCGCGCACCAACAGAGCCGGTTCTCAGGGC

GCTTTGCTCCTTGTTTTTTCCCCGGTTCTGTTTTCTCCCCTTCTCCGGAAGGCTTGTCAA

GGGGTAGGAGAAAGAGACGCAAACACAAAAGTGGAAAACAG↓TTAATGACCAGCCACGGCG

TCCCTGCTGTGAGCTCTGGCCGCTGCCTTCCAGGGCTCCCGAGCCACACGCTGGGGGTGC

TGGCTGAGGGAAC

......(((.((.((((((.(((((((.....(((((...(((..((((.(((.......

..)))...))))..)))...)))))..))))))).)))..((((((((((....(((((.

((...)).)))))........))))))))))((((((((((((....)))))(((....)

))....))))))))..(((.....(((....))).......)))........(((((((((

....))))))).))))).)))))..((((((((.((((((.(((.....))))))))).)

)))..)))))....

>A2_Human

GCGCGGGCGATGCCCAGCGGCGCGGCGGGCTGCGGGGCCCGGCGGGGCGCGCAGAGGAGC

GGGCCGCGGCGCTGAGGCGGCGGAGCGTGGCCCCGCC

(((.((((.((((((.((.((.(((((((((....))))))..((.(((.(((......))

).))).))..)))))..)).)))).)))).)))))))).

>A3_Human

AGTAGCGGGTTGCAGGCGCACCCTCCCCTCCAGGGCGGCCACGCAGCTGTCAGTGCCGCC

GCCACTGCGAGGCTGGAGCGGAGCCCGGGTGGCCGAGGGAGGGGACCCCGCGAGAGGGCC

GCGCGCCGGCCGCCGCCGCCCCGGCGCCCAGGCTCG↓GTGCTGGAGAGTCATGCCTGTGAG

CCCTGGGCACCTCCTGATGTCCTGCGAGGTCACGGTGTTCCCAAACCTCAGGGTTGCCCT

GCCCCACTCCAGAGGCTCTCAGGCCCCACCCCGGAGCCCTCTGTGCGGAGCCGCCTCCTC

CTGGCCAGTTCCCCAGTAGTCCTGAAGGGAGACCTGCTGTGTGGAGCCTCTTCTGGGACC

CAG↓CCATGAGTGTGGAGCTGAGCAACTGAACCTGAAACTCTTCCACTGTGAGTCAAGGAG

GCTTTTCCGCACATGAAGGACGCTGAGCGGGAAGGACTCCTCTCTGCCTGCAGTTGTAGC

GAGTGGACCAGCACCAGGGGCTCTCTAGACTGCCCCTCCTCCATCGCCTTCCCTGCCTCT

CCAGGACAGAGCAGCCACGTCTGCACACCTCGCCCTCTTTACACTCAGTTTTCAGAGCAC

GTTTCTCCTATTTCCTGCGGGTTGCAGCGCCTACTTGAACTTACTCAGACCACCTACTTC

TCTAGCAG↓CACTGGGCGTCCCTTTCAGCAAGACG

rs102978316

rs146642275

rs45518738

rs45487892

(((((..((((..((((((.(((((((((((((((((((...(((........))).)))

))).))).))))..)))).))((((((((((((((.((((((((...((((((((((((.

..(((((((((..(((((...)))))....))).))))))(((...((...(((((.(((

(((((((((((...((((.((....)).)))).)))))..........(((((...))))

).......)))).))))).)))))...)).)))..))))))).)))))..)).)))))).

.))))))...)))..((((......((((((((.((((.(((((..(((.....)))..)

))....(((((((((((....((.............((((.........))))..(((((

.(((((((((.((((.....)).)).))))))))).))))).((((((((.((..((((.

(((.((.........((((((..........))))))..........))))).))))..))

.)))).))))((((......))))........))...))))))))))).....)).)))).

))))).))).....)))))))))...))))))...(((......)))))))..)))))..

(((.((......(((...))).....)).)))..

>A4_Human

GGACACAGCGTCCGGAGCCAGAGGCGCTCTTAACGGCGTTTATGTCCTTTGCTGTCTGAG

GGGCCTCAGCTCTGACCAATCTGGTCTTCGTGTGGTCATTAGC

((((.....))))(((((..(((((.((((((((((((...........)))))).))))

))))))).)))))(((((...))))).................

>A5_Human

ACAGATCCCAGCTGGGTCACCCGCACTGAGTCAACAGACTGAGCGCGTCCAGGCCTGACA

GCTCTGCGGCTCGGGCCCTGAG↓GTTTATTCAGAAAAC

...(((((.....)))))...(((.((.((((....)))).)).)))....(((((((..

(((....))))))))))(((((.....))))).....

>A6_Human

AGAAACCAGGCTGTGTAAGAGCTGCTGGAGTAGGCACCCATTTAAAGAAAAAATGAAGAA

GCAGCAATAAAGAAGTTGTAATCGTTACCTAGACAAACAGAGAACTGGTTTTGACAGTGT

TTCTAGAGTGCTTTTTATTATTTTCCTGACA↓GTTGTGTTCCACCATGATTACTTTCTCCT

TCAGCGAATAGGCTAA

.......((.((((......((((..((((.(((............(((((....(((((

((((((((......)))))....(((.....)))....(((((((((.......)))).)

))))....))))))))....))))).....(((((((......))))))).))).))))).

.))))..))))).))..

>A7_Human

AAGCTCAGCGCACTTGGCTTAAGGGGCGGCGCGCTCCCTGCCTGCTGCTGGGCGGAGGGA

AGGCGGCAAGAGCTGCGGAGCCCCTGGAAG↓AGCTTCCAGGAACCCTGCGCTGTGGGATAA

AGGAATGAGGTTCAGAAAGGGGCAGGGAGTTGCCCGCAGCCGCACCGCACGTCTTCAGCC rs182233998

CGACCGTTGTCCTGACCTCTCTGTCCCGTCCCCTGCCCAGTCTCACC rs3752229

.....(((((.....(((...(((((((...))).))))))))))))((((((((.((((

.((.((((((((((((((.((.((((((((....))))))))..((((((.((...((((..

.........)))).....)).))))))....)))))))))).((...))..(((..((((.

.....))))....))).))).)))))).)))))))))))).......

>A8_Human

ATAACCTCCACTCTGAAAGCAGTCTTCACAGAAACTTTTCACAGAAGTCAAATAGTTAAA

GCAAATTCTAGATACATGGTAGAGACCAGGAGAAAATATGAATAACTTTCTTCTAAACAA

GGAGCTCAGTGGATAAACCATACCTCTAGATTCCTTGCTTCCATTTTCCCAGAA↓GTTTTG

GTAGCAGGATGATGTTGGCCTCATAATGTGAGTTAGAGAGGAGTCCCTCTTTTTCGACTG

TTTGGAATTGTTTCAGAAGGAATGCTACCAGCTCCTCTTGTACCACTGGTAGAATTCAGC

TGTGAATCTGTCTGGTCCTGGGCTTTTTTTGATTG↓ACAAG

........((.((.(((((.(((((...((((.....(((((((....(((((((((.((

((((..((((((...(((((.....(((.(((.................(((((.....)

)))))))..)))....)))))...))))))....))))))........((((..((((((

....))))))....)))).(((((...)))))..(((((((....)))))))...)))))

))))(((....))).....((((.(((((((..............))))))).))))..)

))))))....))))).....))))))))))).)).)).....

>A9_Human

AGAACATGCAGAGACCCATGGATGAACTGTGTTTCTAGATTTTTCCTCCAGCTTTCCTGA

GA↓GAAACAGGTCAAA

((((.((((((...((...)).....)))))))))).(((((((.((((((.....))).

))).)).))))))...

>A10_Human

TAGTAGTACTAACAACGTTTTATAGGAGCACAATTAATTTTACTTAGGATAAGTGTTGTT
ATTATTGTTTTTATTGTTGTTCTGTTAGTTACTCAAAACTTCATTCTAATTGTGCCCTGA
rs563620435
GTTTGTTAAAATACCATACTGTATTTTTGTGTAACATGTAAATAGGCATTAATTTTTGAG
AAATAGAAATGTTTATCCTTAATGTATTTTTAATTTGCTAACATTGATTTTTTATTTTCT
TTCCTGAAATAGCTTATTTCCTAAAATGAAAGAATTTATTCTCAGATGAATAATTTTTAT
ATCAGCTATTCTTATCAG↓AGCAATAAACAAATACCAATGATGCGCTCAGCCAACAATTCA
TTACACTCTCTGAAGAGTAACTGGACAAGGAGAAAAACATAGGGAAAAAACCAACAGAAT
rs1238052530
TTGTTGGCATGTTCTACACACAGACCATGGCTTTTCAGAAGCCAAGCTGAATCAAAACAG
rs9302891
TTTTAAAAGAGGCAACCATTTGTAGAGGAGTCCTTGAAGGATTCTTCATTGTTTTCTTGG
ACAAAAAGAGACCAGTGGATCCAAGTGCTTCAAATACTTCTCTCTTATTTTCTTAACT↓CT
ATTGCTCTGCAATATTTACTTTACCCTGTTAATGAACAGGACAAAATGGTTAAAAAAGAG
ATAAGCGTGCGTCAACAAATTCAGGCTCTTCTGTACAAGAATTTTCTTAAAAAATGGAGA
ATAAAAAGAGAGTTTATTG↓GAATGGACAATAACATTGTTTCTAGGGCTATATTTGTGCAT
rs1024510317
CTTTTCGGAACACTTCAGAGCTACCCGTTTTCCTGAACAACCTCCTAAAGTCCTGGGAAG
CGTGGATCAGTTTAATGACTCTGGCCTGGTAGTGGCATATACACCAGTCAGTAACATAAC
ACAAAGGATA

................(((((..((((((((((.((......((((((..(((((((((
(((((((.(((..(((((((((((.(((...((((((..........((((((((....
...(((((((((((......)))))).....)))))........)))).)))))))))))
..(((((((((((((((.(((((((...............))))))).....((..((((
(((..((((((...))))))).......)))))))..))......))))))).))))))))
...........))).)))))))))))..)))...)))))))((.....))...((.((((
.((.((((((((((((((..((.....))............((.......))....((((
(((((((((((((((.......((((((((((((...))))))((((((........)))
)))....((((............((((((((..((((((.(((.(((((((.((((((..
.....)))))).))))))).....))).))))))..))))))))............)))))
(((((...))))).............((((((....))))))......)))))).......))
)...)))))))..))))))))))..)))))))).........(((((((........)))))
))....))))))).)).)))).))...)))))))))..)))))))....)).)))))).)
)))...))))).......(((((.((((((((((...((..........))...)))))))
)).))...))))).(((...(((((.((((.(((.....))))))))))))...)))...
....

>A12_Human

GTGGATAGGCAATTAATTTCATGGAGAGTAGGAGGTGTGGGTTCAGCTGCTGCTTTTTTT
TTTTTTTCAGTGAGGCATTATCTGAATGATGCTCATTCTCTCTTGAGTCTGGACTTCGTG
CAACAAGCAGCAACTGGATATTATTTTAAAATAAGCATAAGCCACCCAAAGATACTGATC
TGGGTCCTTCTTTTGGAAGAAGAGTTGATTGAGAAGTGCCTCTTGGTTAAGGATTAACCA
CAGGGAAAAATCCAGCAGAAACAGAAGAACTGTGGGTTTCTTACCCCAGCCCTCAAGGAA
GCTATGCCGTGAAAGGGGTACTGATACACTGACATACAGCAAGTTGGACGGGGCATCAGT
TCTTCATTTGTGGAGTGGAGAAAAGAAGAGGAAATCTCTCATTTGGGGCATTTGAAGG

.........((((((((((....(((((.((((((.((((((((((.((((((((.....
........((((((.((((((....))))))))))))(((....)))..((.((...)).
))..)))))))).)))))).(((((....))))).......))))))..((((....)))
)...)))).))))).......))))))))))..(((((((((.(((((((...)))))))
...(((....)))..((.(((..(((((((((.((((.....))))..(((((......(
(((.(((.(((....(((((....))).))....))).))))))).....))))).))))
))))).))).))(((((((((.............)))).)))))))))))))).....

>A13_Human

CTGACTGAGAGCAGGGAGCAGCAGGC

(((.(((...........))))))..
