# Supplementary material for: 5′ Untranslated Region Elements Show High Abundance and Great Variability in Homologous ABCA Subfamily Genes
Source: Int J Mol Sci. 2020 Nov 23;21(22):8878. doi: 10.3390/ijms21228878 (PMC7700387; doi:10.3390/ijms21228878)
Supplement: Supplementary file 1 [file ijms-21-08878-s001.zip › Supplementary Information Tables v3 Dvorak.pdf]

Table S1: Basic characteristics of the transcripts downloaded and analyzed in the study (Ensembl release 99 - January 2020 © EMBL-EBI).

Gene/ No. of Tr.	Species (Assembly)	Tr. Name	Tr. ID	Tr. Length (bp)	Protein Length (aa)
ABCA1					
1	Human (GRCh38.p13)	ABCA1-202	ENST00000374736.8	10408	2261aa
2	Marmoset (ASM275486v1)	ABCA1-203	ENSCJAT00000082284.2	7069	2256aa
3	Angola colobus (Cang.pa_1.0)	ABCA1-201	ENSCANT00000035942.1	10490	2261aa
4	Golden Hamster (MesAur1.0)	Abca1-201	ENSMAUT00000018446.1	10229	2261aa
5	Mouse (GRCm38.p6)	Abca1-201	ENSMUST00000030010.3	10262	2261aa
6	Arabian camel (CamDro2)	ABCA1-201	ENSCDRT00005020754.1	8207	2261aa
7	Canada lynx (mLynCan4_v1.p)	ABCA1-201	ENSLCNT00005022774.1	7890	2261aa
8	Armadillo (Dasnov3.0)	ABCA1-201	ENSDNOT00000009223.3	7192	2264aa
9	Emu (droNov1)	ABCA1-201	ENSDNVT00000000545.1	7036	2261aa
10	Three-toed box turtle (T_m_triunguis-2.0)	ABCA1-201	ENSTMTT00000004136.1	8530	2200aa
11	Coelacanth (LatCha1)	ABCA1-201	ENSLACT00000009960.1	5299	1525aa
ABCA2					
1	Human (GRCh38.p13)	ABCA2-202	ENST00000341511.11	8103	2436aa
2	Capuchin (Cebus_imitator-1.0)	ABCA2-202	ENSCCAT00000050655.1	8097	2434aa
3	Sooty mangabey (Caty_1.0)	ABCA2-201	ENSCATT00000064419.1	8417	2549aa
4	Golden Hamster (MesAur1.0)	Abca2-201	ENSMAUT00000001022.1	8185	2434aa
5	Mouse (GRCm38.p6)	Abca2-201	ENSMUST00000102919.3	8033	2433aa
6	Ferret (MusPutFur1.0)	ABCA2-201	ENSMPUT00000010911.1	9291	2365aa
7	Donkey (ASM303372v1)	ABCA2-201	ENSEAST00005027735.1	8803	2748aa
8	Painted turtle (Chrysemys_picta_bellii-3.0.3)	ABCA2-201	ENSCPBT00000017126.1	7756	2480aa
9	Duck (CAU_duck1.0)	ABCA2-201	ENSAPLT00000036122.1	10196	2437aa
10	Anole lizard (AnoCar2.0)	ABCA2-201	ENSACAT00000007708.3	7435	2393aa
11	Denticle herring (fDenClu1.1)	abca2-201	ENSDCDT00000031988.1	8822	2512aa
ABCA3					
1	Human (GRCh38.p13)	ABCA3-201	ENST00000301732.10	6602	1704aa
2	Angola colobus (Cang.pa_1.0)	ABCA3-201	ENSCANT00000012088.1	6448	1704aa
3	Capuchin (Cebus_imitator-1.0)	ABCA3-201	ENSCCAT00000057064.1	6811	1703aa
4	Guinea Pig (Cavpor3.0)	ABCA3-201	ENSCPOT00000038719.1	6790	1704aa
5	Mouse (GRCm38.p6)	Abca3-201	ENSMUST00000039013.14	6476	1704aa
6	Leopard (PanPar1.0)	ABCA3-202	ENSPPRT00000016709.1	6348	1704aa
7	Arabian camel (CamDro2)	ABCA3-201	ENSCDRT00005025167.1	5970	1703aa
8	Armadillo (Dasnov3.0)	ABCA3-201	ENSDNOT00000050172.1	10886	1728aa
9	Painted turtle (Chrysemys_picta_bellii-3.0.3)	ABCA3-201	ENSCPBT00000000241.1	10045	1707aa
10	Bengalese finch (LonStrDom1)	ABCA3-201	ENSLSDT00000001885.1	7684	1708aa
11	Komodo dragon (ASM479886v1)	ABCA3-201	ENSVKKT00000016923.1	5639	1708aa
ABCA4					
1	Human(GRCh38.p13)	ABCA4-201	ENST00000370225.4	7328	2273aa
2	Angola Colobus(Cang.pa_1.0)	ABCA4-201	ENSCANT00000061435.1	7323	2273aa
3	Tarsier(Tarsius_syrichta-2.0.1)	ABCA4-201	ENSTSYT00000013324.2	6965	2273aa
4	Mouse(GRCm38.p6)	Abca4-201	ENSMUST00000013995.12	7263	2310aa
5	Degu(OctDeg1.0)	ABCA4-201	ENSODET00000007042.1	7064	2326aa
6	Cow(ARS-UCD1.2)	ABCA4-201	ENSBTAT00000023982.6	7708	2281aa
7	Pig (Sscrofa11.1)	ABCA4-201	ENSSSCT00000007545.4	7084	2320aa
8	Dog (CanFam3.1)	ABCA4-201	ENSCAFT00000005367.5	7014	2321aa
9	Painted Turtle(Chrysemis_picta_bellii-3.0.3)	ABCA4-201	ENSCPBT00000050117.1	7340	2332aa
10	Chicken(GRCg6a)	ABCA4-201	ENSGALT00000009238.5	6839	2272aa
11	Yellowtail amberjack (Sedor1)	abca4a-201	ENSSLDT00000010988.1	9812	2271aa
ABCA5					
1	Human (GRCh38.p13)	ABCA5-201	ENST00000392676.8	8252	1642aa
2	Vervet-AGM (ChlSab1.1)	ABCA5-201	ENSCSAT00000002404.1	8510	1642aa
3	Ugandan red Colobus (ASM277652v2)	ABCA5-201	ENSPTET00000004315.1	5314	1642aa
4	Mouse (GRCm38.p6)	Abca5-201	ENSMUST00000043961.11	8231	1642aa
5	Golden Hamster (MesAur1.0)	Abca5-201	ENSMAUT00000013769.1	8811	1642aa
6	Cow (ARS-UCD1.2)	ABCA5-201	ENSBTAT00000003563.6	5791	1562aa
7	Canada lynx (mLynCan4_v1.p)	ABCA5-201	ENSLCNT00005037861.1	5268	1642aa
8	Great spotted kiwi (aptHaa1)	ABCA5-201	ENSAHAT00000029191.1	5076	1646aa
9	Chicken (GRCg6a)	ABCA5-201	ENSGALT00000006907.6	5164	1647aa
10	Australian saltwater crocodile (CroPor_comp1)	ABCA5-201	ENSCPRT00005031181.1	5486	1646aa
11	Agassiz's desert tortoise (ASM289641v1)	ABCA5-201	ENSGAGT00000040318.1	5018	1646aa
ABCA6					
1	Human (GRCh38.p13)	ABCA6-201	ENST00000284425.7	5321	1617aa
2	Bolivian squirrel monkey (SaiBol1.0)	ABCA6-201	ENSSBOT00000048276.1	5912	1631aa
3	Vervet-AGM (ChlSab1.1)	ABCA6-201	ENSCSAT00000002382.1	5560	1618aa
4	Golden snub-nosed monkey (Rrox_v1)	ABCA6-201	ENSRROT00000016366.1	5427	1618aa
5	Squirrel (SpeTri2.0)	ABCA6-201	ENSSTOT00000013298.3	6087	1629aa
6	Rabbit (OryCun2.0)	ABCA6-201	ENSOCUT00000014391.4	5012	1618aa
7	Mouse (GRCm38.p6)	Abca6-201	ENSMUST00000044003.13	5240	1624aa
8	Guinea Pig (Cavpor3.0)	ABCA6-201	ENSCPOT00000042666.1	6044	1465aa
9	Meerkat (meerkat_22Aug2017_6uvM2_HiC)	ABCA6-201	ENSSSUT00005033782.1	5383	1616aa
10	Ferret (MusPutFur1.0)	ABCA6-201	ENSMPUT00000014462.1	5226	1621aa
11	Arabian camel (CamDro2)	ABCA6-201	ENSCDRT00005031706.1	5494	1576aa

No., Number; Tr., Transcript;

Table S1: Continued.

Gene/ No. of Tr.	Species (Assembly)	Tr. Name	Tr. ID	Tr. Length (bp)	Protein Length (aa)
ABCA7					
1	Human (GRCh38.p13)	ABCA7-201	ENST00000263094.11	6815	2146aa
2	Vervet-AGM (ChlSab1.1)	ABCA7-201	ENSCSAT00000010455.1	6768	2149aa
3	Macaque (Mmul_10)	ABCA7-202	ENSMMUT00000021458.4	6830	2111aa
4	Bolivian squirrel monkey (SaiBol1.0)	ABCA7-201	ENSSBOT00000024453.1	7030	2144aa
5	Mouse (GRCm38.p6)	Abca7-202	ENSMUST00000132517.7	6696	2159aa
6	Prairie vole (MicOch1.0)	Abca7-201	ENSMOCT00000014061.1	6688	2161aa
7	Pig (Sscrofa11.1)	ABCA7-201	ENSSSCT00000061946.2	6978	2206aa
8	Ferret (MusPutFur1.0)	ABCA7-201	ENSMPUT00000009091.1	6769	2152aa
9	Goat (ARS1)	ABCA7-201	ENSCHIT00000035014.1	6583	2152aa
10	Painted turtle (Chrysemys_picta_bellii-3.0.3)	ABCA7-201	ENSCPBT00000015380.1	3646	1100aa
11	Spotted gar (LepOcu1)	zgc:172302-201	ENSLOCT00000002958.1	6925	2237aa
ABCA8					
1	Human (GRCh38.p13)	ABCA8-208	ENST00000586539.6	6002	1621aa
2	Bolivian squirrel monkey (SaiBol1.0)	ABCA8-202	ENSSBOT00000054772.1	7060	1621aa
3	Macaque (Mmul_10)	ABCA8-201	ENSMMUT00000078307.2	5789	1621aa
4	Mouse Lemur (Mmur_3.0)	ABCA8-201	ENSMICT00000039031.2	5815	1623aa
5	Vervet-AGM (ChlSab1.1)	ABCA8-201	ENSCSAT00000002281.1	5115	1623aa
6	Long-tailed chinchilla (ChiLan1.0)	ABCA8-202	ENSCLAT00000005785.1	9636	1621aa
7	Rabbit (OryCun2.0)	ABCA8-201	ENSOCUT00000008019.4	5036	1596aa
8	Mouse (GRCm38.p6)	Abca8b-201	ENSMUST00000020948.14	7423	1620aa
9	Red fox (VulVul2.2)	ABCA8-203	ENSVVUT00000013657.1	5836	1622aa
10	Meerkat (meerkat_22Aug2017_6uvM2_HiC)	ABCA8-201	ENSSSUT00005032074.1	5804	1623aa
11	Pig (Sscrofa11.1)	ABCA8-205	ENSSSCT00000054209.2	5560	1566aa
ABCA9					
1	Human (GRCh38.p13)	ABCA9-201	ENST00000340001.9	6377	1624aa
2	Angola colobus (Cang.pa_1.0)	ABCA9-201	ENSCANT00000048814.1	6543	1624aa
3	Drill (Mleu.le_1.0)	ABCA9-201	ENSMLET00000054252.1	5631	1624aa
4	Mouse Lemur (Mmur_3.0)	ABCA9-201	ENSMICT00000032785.2	5716	1625aa
5	Long-tailed chinchilla (ChiLan1.0)	ABCA9-201	ENSCLAT00000011024.1	8852	1624aa
6	Kangaroo rat (Dord_2.0)	Abca9-201	ENSDORT00000003286.2	5616	1629aa
7	Prairie vole (MicOch1.0)	Abca9-201	ENSMOCT00000019981.1	6685	1624aa
8	Mouse (GRCm38.p6)	Abca9-201	ENSMUST00000044850.3	6384	1623aa
9	Pig (Sscrofa11.1)	ABCA9-201	ENSSSCT00000023395.3	6543	1623aa
10	Canada lynx (mLynCan4_v1.p)	ABCA9-201	ENSLCNT00005034192.1	7010	1625aa
11	Armadillo (Dasnov3.0)	ABCA9-202	ENSDNOT00000033964.1	9505	1626aa
ABCA10					
1	Human (GRCh38.p13)	ABCA10-201	ENST00000269081.8	6362	1543aa
2	Angola colobus (Cang.pa_1.0)	ABCA10-201	ENSCANT00000059450.1	6361	1545aa
3	Meerkat (meerkat_22Aug2017_6uvM2_HiC)	ABCA10-201	ENSSSUT00005034007.1	5313	1486aa
4	Canada lynx (mLynCan4_v1.p)	ABCA10-201	ENSLCNT00005037701.1	5323	1368aa
5	Arabian camel (CamDro2)	ABCA10-201	ENSCDRT00005030804.1	5715	1613aa
6	Wild yak (BosGru_v2.0)	ABCA10-201	ENSBMUT00000035871.1	5855	1630aa
7	Common canary (SCA1)	ABCA10-201	ENSSCAT00000020843.1	4992	1539aa
8	White-throated sparrow (Zonotrichia_albicollis-1.0.1)	ABCA10-201	ENSZALT00000008796.1	4981	1462aa
ABCA12					
1	Human (GRCh38.p13)	ABCA12-201	ENST00000272895.12	9298	2595aa
2	Pig-tailed macaque (Mnem_1.0)	ABCA12-201	ENSMNET00000037858.1	9239	2595aa
3	Marmoset (ASM275486v1)	ABCA12-201	ENSCJAT00000022637.4	8233	2595aa
4	Rabbit (OryCun2.0)	ABCA12-201	ENSOCUT00000044085.1	8127	2577aa
5	Prairie vole (MicOch1.0)	Abca12-201	ENSMOCT00000019649.1	8349	2596aa
6	Mouse (GRCm38.p6)	Abca12-201	ENSMUST00000087268.6	9137	2595aa
7	Long-tailed chinchilla (ChiLan1.0)	ABCA12-201	ENSCLAT00000009751.1	8872	2598aa
8	Red fox (VulVul2.2)	ABCA12-201	ENSVVUT00000015996.1	8674	2597aa
9	Pig (Sscrofa11.1)	ABCA12-201	ENSSSCT00000042475.2	9336	2593aa
10	Chinese softshell turtle (PelSin_1.0)	ABCA12-201	ENSPSIT00000017400.1	8344	2585aa
11	African ostrich (ASM69896v1)	ABCA12-202	ENSSCUT00000023862.1	6601	2122aa
ABCA13					
1	Human (GRCh38.p13)	ABCA13-204	ENST00000435803.6	17188	5058aa
2	Angola colobus (Cang.pa_1.0)	ABCA13-201	ENSCANT00000056247.1	15590	5057aa
3	Mouse (GRCm38.p6)	Abca13-201	ENSMUST00000042740.12	16011	5034aa

No., Number; Tr., Transcript;

Table S2: A detailed overview of 5'UTR features in the human ABCA genes.

Gene Abbr.	5'UTR length [nt]	No. of uATGs	uATG pos.	uATG cons.	uATG fl. seq. context	uATG TIS score	No. of uORFs	uORF start	uORF end	uORF length [nt]	Ribo-seq cov.	sATG fl. seq. context
**A3**	694	4	-525	3	2	0.718	2	-525	-376	150	3	3
			-498	4	1	0.629						
			-329	1	2	0.367						
			-262	1	1	0.368		-262	>+1	>261	2	
**A1**	313	1	-89	5	1	0.187	1	-89	>+1	>87	3	3
**A4**	103	1	-62	3	1	0.047	0					3
**A5**	97	0					0					2
**A10**	910	14	-755	1	2	0.217	6					2
			-722	1	2	0.379		-722	-690	33	1	
			-709	1	1	0.19						
			-645	4	2	0.529						
			-625	1	1	0.306		-625	-509	117	1	
			-574	1	1	0.187						
			-571	0	2	0.311						
			-482	1	2	0.355		-482	-441	42	1	
			-464	3	3	0.402						
			-279	3	1	0.175		-279	-247	33	1	
			-265	3	3	0.625		-265	-95	171	1	
			-197	3	3	0.46		-197	-159	39	1	
			-169	3	3	0.323						
			-56	3	1	0.074						
**A6**	196	2	-144	3	2	0.356	0					1
			-32	1	2	0.102						
**A12**	418	4	-398	1	2	0.233	1	-398	-330	69	1	3
			-333	4	1	0.306						
			-330	1	2	0.279						
			-115	4	2	0.31						
**A13**	26	0					0					3
**A9**	75	3	-70	3	2	0.1	2	-70	-38	33	1	2
			-58	1	2	0.098						
			-54	3	1	0.056		-54	-16	39	1	
**A8**	340	6	-265	0	2	0.206	2					2
			-243	1	2	0.547		-243	-205	39	3	
			-152	0	2	0.211						
			-149	0	2	0.285						
			-135	0	2	0.362						
			-79	0	2	0.085		-79	-5	75	1	
**A7**	227	1	-103	3	2	0.359	1	-103	>+1	>102	3	3
**A2**	97	1	-88	2	2	0.108	1	-88	-23	66	1	3

Abbr., abbreviation; Cons., conservation/conserved; Cov., coverage; Fl., flanking; Fo., forming; No., number; Pos., position (from sATG); Reg., region; sATG, start ATG of the main ORF; Seq., sequence; St., stem; TSS, transcription start site; TIS, translation initiation start; uATG, upstream ATG

uATG conservation: 0=Human, 1=Primates, 2=Pri + Rodents, 3=Placental mammals, 4=Plac. mam. + Reptiles and birds, 5=Plac. mam. + Rep. and bi. + Coelacanth/ray-finned fishes; **uATG/sATG flanking sequence context:** 1) weak (NNN(C/U)NNAUG(A/C/U), 2) adequate (NNN(A/G)NNAUG(A/C/U) or NNN(C/U)NNAUGG, 3) strong (NNN(A/G)NNAUGG, 4) optimal (GCC(A/G)CCAUGG), (Hernandez et al., 2019); **uATG TIS score (NetStart software):** The scores are in [0.0, 1.0]; when greater than 0.5 they represent a probable translation start; **Ribo-seq coverage:** 1=Low, 2=Medium, 3=High

Table S2: Continued.

Gene Abbr.	Most cons. reg. pos.	No. of 5'UTR introns	Intron pos.	Intron length [nt]	RG4-fo. seq. pos.	No. of 5'UTR st. loops	No. of all 5'UTR elements
**A3**	-519.-503	3	-539/538<br>-332/331<br>-27/26	10718<br>890<br>1960	-608.-576	5	13
**A1**	-119.-59	1	-93/92	24163	-251.-218	6	9
**A4**	-65.-50	0		0		2	3
**A5**	-7.-2	1	-16/15	12621		4	5
**A10**	-459.-368	3	-593/592<br>-313/312<br>-172/171	15668<br>1295<br>1615		5	22
**A6**	-156.-63	1	-46/45	996		3	6
**A12**	-342.-318<br>-269.-231<br>-120.-102	0		0		6	10
**A13**		0		0		0	0
**A9**	-75.-60<br>-46.-14	1	-14/13	9726		2	6
**A8**	-234.-180	2	-167/166<br>-6/5	5746<br>7272		3	11
**A7**	-137.-73	1	-138/137	1028	-217.-158	3	6
**A2**	-65.-12	0		0	-81.-34	2	4

Abbr., abbreviation; Cons., conservation/conserved; Cov., coverage; Fl., flanking; Fo., forming; No., number; Pos., position (from sATG); Reg., region; sATG, start ATG of the main ORF; Seq., sequence; St., stem; TSS, transcription start site; TIS, translation initiation start; uATG, upstream ATG

uATG conservation: 0=Human, 1=Primates, 2=Pri + Rodents, 3=Placental mammals, 4=Plac. mam. + Reptiles and birds, 5=Plac. mam. + Rep. and bi. + Coelacanth/ray-finned fishes; **uATG/sATG flanking sequence context:** 1) weak (NNN(C/U)NNAUG(A/C/U), 2) adequate (NNN(A/G)NNAUG(A/C/U) or NNN(C/U)NNAUGG, 3) strong (NNN(A/G)NNAUGG, 4) optimal (GCC(A/G)CCAUGG), (Hernandez et al., 2019); **uATG TIS score (NetStart software):** The scores are in [0.0, 1.0]; when greater than 0.5 they represent a probable translation start; **Ribo-seq coverage:** 1=Low, 2=Medium, 3=High

Table S3: Correlation tables of the first correlation analysis with Spearman's rs and Kendall's tau coefficients and relevant p values

Sperman rs /p value	5'U. length	No. uATGs	sATG cont.	No. introns	Int. length	No. RG4s	No. st. loops	Prot. distrib.	Prot. expres.
5'U. length		0.004	0.975	0.037	0.074	0.577	0.002	0.283	0.166
No. uATGs	0.759		0.305	0.041	0.216	0.626	0.174	0.348	0.338
sATG cont.	0.010	-0.324		0.119	0.336	0.046	0.975	0.226	0.749
No. introns	0.606	0.596	-0.475		0.000	0.737	0.145	0.832	0.388
Int. length	0.533	0.385	-0.304	0.871		0.463	0.042	0.411	0.818
No. RG4s	0.180	-0.157	0.584	0.108	0.235		0.515	0.925	0.448
No. st. loops	0.792	0.420	0.010	0.447	0.592	0.209		0.530	0.640
Prot. distrib.	-0.355	-0.314	-0.398	0.073	0.276	0.032	0.213		0.667
Prot. expres.	-0.449	-0.319	0.109	-0.289	-0.079	-0.256	-0.159	0.147	

Kendall tau /p value	5'U. length	No. uATGs	sATG cont.	No. introns	Int. length	No. RG4s	No. st. loops	Prot. distrib.	Prot. expres.
5'U. length		0.006	1.000	0.016	0.050	0.487	0.004	0.263	0.191
No. uATGs	0.603		0.253	0.022	0.145	0.528	0.138	0.235	0.235
sATG cont.	0.000	-0.252		0.062	0.303	0.010	0.924	0.126	0.724
No. introns	0.532	0.506	-0.412		0.000	0.648	0.062	0.748	0.335
Int. length	0.432	0.322	-0.227	0.793		0.353	0.011	0.296	0.775
No. RG4s	0.153	-0.139	0.566	0.101	0.205		0.401	0.897	0.300
No. st. loops	0.635	0.328	0.021	0.413	0.559	0.186		0.424	0.617
Prot. distrib.	-0.261	-0.277	-0.357	0.075	0.244	0.030	0.187		0.510
Prot. expres.	-0.305	-0.277	0.082	-0.225	-0.067	-0.242	-0.117	0.154	

Abbreviations: 5'U., 5'UTR; Cont., context; Distrib., distribution; Expres., expression; Int., intron; No., number; Prot., protein; RG4, RNA G-quadruplex; sATG, start ATG of the main ORF; St., stem; uATG, upstream ATG.

Table S4: Correlation tables of the second correlation analysis with Spearman's rs and Kendall's tau coefficients and relevant p values

Spearman rs/ p value	uATG pos.	uATG cons.	uATG cont.	uATG TIS sc.
uATG pos.		0.632	0.543	0.034
uATG cons.	0.081		0.386	0.366
uATG cont.	0.103	-0.147		0.011
uATG TIS sc.	0.350	0.153	0.414	

Kendall tau/ p value	uATG pos.	uATG cons.	uATG cont.	uATG TIS sc.
uATG pos.		0.629	0.507	0.031
uATG cons.	0.055		0.277	0.341
uATG cont.	0.076	-0.125		0.003
uATG TIS sc.	0.248	0.109	0.341	

Abbreviations: uATG pos., uATG position (from transcription start site); uATG cons., uATG conservation; uATG cont., uATG flanking sequence context; uATG TIS sc., uATG TIS score (from NetStart);
