# Supplementary figures and images for: 5′ Untranslated Region Elements Show High Abundance and Great Variability in Homologous ABCA Subfamily Genes
Source: Int J Mol Sci. 2020 Nov 23;21(22):8878. doi: 10.3390/ijms21228878 (PMC7700387; doi:10.3390/ijms21228878)

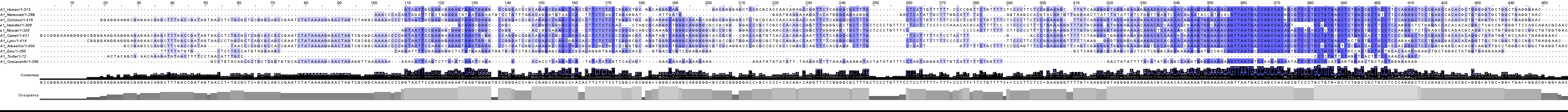

Supplement: Supplementary file 1 [file ijms-21-08878-s001.zip › Figure S1 v1 A1 ClustalO.jpg]

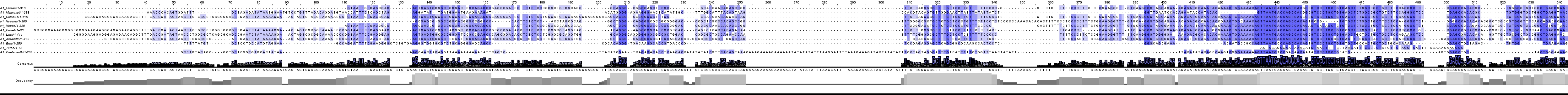

Supplement: Supplementary file 1 [file ijms-21-08878-s001.zip › Figure S2 v1 A1 Mafft.jpg]

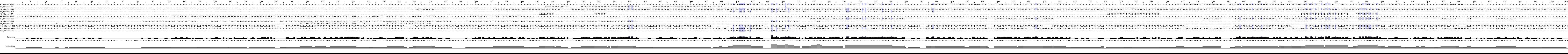

Supplement: Supplementary file 1 [file ijms-21-08878-s001.zip › Figure S3 v1 Aall ClustalO.jpg]

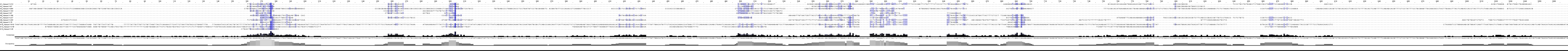

Supplement: Supplementary file 1 [file ijms-21-08878-s001.zip › Figure S4 v1 Aall Mafft.jpg]
